# Diagnostic performance of dual-layer spectral CT Radiomics and deep learning for differentiating osteoblastic bone metastases from bone islands

**DOI:** 10.1016/j.ejro.2025.100679

**Published:** 2025-08-20

**Authors:** Yuchao Xiong, Wei Guo, Xuwen Zeng, Fan Xu, Li Wu, Jiahui Ou

**Affiliations:** a Department of Radiology, Guangzhou Red Cross Hospital (Guangzhou Red Cross Hospital of Jinan University), 396 Tongfu road, Guangzhou, Guangdong Province 510220, China; b Department of Radiology, Wuhan Third Hospital, Tongren Hospital of Wuhan University, 241 Liuyang Road, Wuhan, Hubei Province 430063, China

**Keywords:** Computed tomography, Deep learning, Dual-energy CT, Bone neoplasms

## Abstract

**Background:**

This study aimed to compare the diagnostic performance of radiomic features derived from dual-layer spectral detector computed tomography (DLSCT) and a deep learning (DL) model applied to conventional CT images in the differentiation of osteoblastic bone metastases (OBM) from bone islands (BI).

**Methods:**

This retrospective study included patients with osteogenic lesions who underwent DLSCT examinations between March 2023 and September 2023. We extracted first-order radiomic features (e.g., mean, maximum, entropy) from both conventional and spectral images. A previously validated DL model was applied to the conventional CT images. We evaluated diagnostic performance using ROC curve analysis, comparing AUC, sensitivity, and specificity.

**Results:**

The study included 216 lesions from 94 patients (66 ± 12 years; 48 males, 46 females): 125 BI and 91 OBM lesions. Significant differences were observed between OBM and BI groups for the mean, maximum, entropy, and uniformity of first-order radiomic features (all P < 0.05). DLSCT (entropy from VMI40keV) and the DL model had comparable AUCs (0.93 vs. 0.96; P = 0.274). However, DLSCT showed superior sensitivity (92 % vs. 62 %; P = 0.002) but comparable specificity (88 % vs. 96 %; P = 0.07) for diagnosing OBM compared to the DL model.

**Conclusion:**

Radiomic features from DLSCT differentiate between BI and OBM with diagnostic performance comparable to that of a DL model. Furthermore, VMI40keV image-derived entropy demonstrated superior sensitivity in diagnosing OBM compared to the DL model.

## Highlights

• DLSCT & DL: New tools to distinguishing BI from OBM.
• DLSCT matches DL, but has better sensitivity.
• Study compares DLSCT & DL: aids method choice.

## 1 Introduction

Sclerotic bone lesions are frequently encountered on computed tomography (CT) images, with a reported incidence exceeding 3.2 %, a figure that has risen concurrently with the widespread adoption of CT examinations [1], [2]. The differentiation of these lesions as either bone islands (BI) or osteoblastic bone metastases (OBM) are critical, as it dictates subsequent clinical management. This distinction is often complicated in patients with a history of malignancy, as treatments like chemotherapy and bisphosphonates can induce osteoblastic repair[3], [4], [5], [6]. This repair alters lesion appearance on conventional CT, making it difficult to distinguish OBM from benign BI. Patients diagnosed with BI typically do not require further intervention, whereas those with OBM necessitate a comprehensive investigation to identify the primary malignancy, determine tumor stage, and establish appropriate therapeutic strategies. Therefore, rapid and accurate discrimination between OBM and BI is paramount for radiologists to minimize diagnostic delays, enhance diagnostic precision, and guide optimal patient management.

Deep learning (DL), a subfield of machine learning, has demonstrated considerable success in computer vision applications. Convolutional Neural Networks (CNNs), the cornerstone of DL methods in image analysis, have found numerous applications in radiology, particularly in image classification tasks [7]. Previous research has demonstrated the excellent performance of CNN-based DL models in differentiating BI and OBM using CT images [8]. These models autonomously generate diagnostic outputs by processing three consecutive image slices containing the lesion [8]. However, the efficacy of these DL models has not been evaluated using images reconstructed from spectral CT acquisitions.

Dual-Energy CT (DECT), employing energy-dependent information inherent in CT data, represents a conceptually straightforward yet powerful imaging modality [9]. DECT provides both high-resolution anatomical detail and valuable quantitative parameters [10]. Prior studies have shown that the effective atomic number derived from dual-tube DECT [11] and 140 kVp images from single-tube rapid kV-switching DECT [12] can aid in distinguishing between BI and OBM. While results have been somewhat inconsistent, these findings suggest the potential of DECT-derived parameters for improved BI and OBM differentiation. Dual-layer spectral CT (DLSCT), a specific DECT implementation, offers a distinct advantage over dual-tube and rapid kV-switching systems by acquiring spectral data in a single exposure, thus avoiding increased radiation dose or scan time [13]. To date, however, the diagnostic utility of DLSCT in differentiating BI from OBM remains unexplored.

Both DL and DLSCT hold significant promise for enabling rapid and efficient diagnosis of BI and OBM without subjecting patients to additional radiation exposure or prolonged scan times [9], [12], [14]. Nevertheless, no prior investigations have directly compared the diagnostic performance of DL and spectral CT parameters in this specific clinical context.

To address this gap, our study was designed as a direct comparison of two distinct and relatively novel diagnostic approaches: 1) the quantitative analysis of first-order radiomic features derived from DLSCT and 2) a DL model applied to conventional CT images. This allowed us to determine if DLSCT's spectral information or a DL model's advanced pattern recognition offers a superior method for differentiating BI and OBM. We also conducted secondary comparisons, evaluating the performance of first-order radiomic features from DLSCT against those from conventional CT, and comparing the DL model's performance to that of conventional CT first-order radiomics.

## 2 Materials and methods

### 2.1 Patients

This retrospective study was approved by the institutional review board of Guangzhou Red Cross Hospital, and the requirement for informed consent was waived. Patients who underwent spectral CT examinations between March 21, 2023, and September 30, 2023, were considered for inclusion. A retrospective review was conducted of 2135 spectral CT images from 1909 patients acquired during this period.

The inclusion criteria were: (1) presence of lesions identifiable on three consecutive CT slices, (2) absence of severe motion artifacts on images, and (3) absence of metallic artifacts that would compromise lesion assessment. The exclusion criterion was the unavailability of complete spectral multiparameter data.

Lesion diagnosis was determined by two independent radiologists based on a combination of patient clinical history and follow-up imaging. BIs were diagnosed based on the absence of a prior history of malignancy and confirmed by the lack of change in size, shape, or density on a follow-up CT scan obtained at least 6 months prior to the current examination [4], [15], [16]. OBMs were diagnosed based on a documented history of a primary malignant tumor (lung or breast cancer) and the presence of hyperdense bone lesions that were either newly identified on the current examination or had demonstrated interval progression (e.g., increased size or density) on follow-up imaging[15]. We acknowledge that histopathological confirmation was not available for all cases, which is a common limitation in retrospective studies of osteogenic lesions [17].

### 2.2 CT protocol

All examinations were performed using a contrast-enhanced dual-layer spectral detector CT scanner (Spectral CT 7500, Philips Healthcare). This system provides both conventional (120 kVp) and spectral-based images from a single acquisition, eliminating the need for prospective scan mode selection [18]. The CT acquisition parameters were as follows: 120 kVp, 50–350 mAs with automatic tube current modulation, and a section thickness of 3 mm. Images were reconstructed axially with a slice thickness of 1.0 mm. A detailed summary of the acquisition protocol is provided **in** Supplementary Table 1**.**

All participants were examined using a contrast-enhanced dual-layer spectral detector CT (spectral CT 7500, Philips Healthcare), which provided conventional (120 kVp) and spectrum-based images for examinations, eliminating the need for prospective selection. The CT protocols were as follows: 120 kVp, 50–350 mAs with automatic tube current modulation, and 3 mm section thickness. The images were reconstructed axially with a slice thickness of 1.0 mm. The detailed protocol is summarized in Supplementary Table1.

Conventional 120-kVp poly-energetic images, generated using an iterative reconstruction algorithm, were archived to the Picture Archiving and Communication System (PACS). Other spectral-based imaging datasets were generated using a proprietary workstation (IntelliSpace Portal, version 9.0; Philips Healthcare) for post-processing, enabling the acquisition of virtual monoenergetic images (VMI) ranging from 40 to 200 keV (VMI 40–200 keV).

### 2.3 Image post-processing and data measurement

All CT images were retrospectively and independently interpreted by two radiologists (YC.X **and** F.X**)**, with 8 and 11 years of experience in musculoskeletal radiology, respectively. Their first task was to establish the definitive diagnosis for each lesion based on patient history and follow-up, which served as the gold standard. Their second task was to perform image annotations for the subsequent analyses.

Conventional 120-keV images generated by spectral CT were retrieved from the PACS and imported into the open-source ITK-SNAP software (version 3.8.0; www.itk-snap.org).The ITK-SNAP software was employed to delineate the region of interest (ROI), which was defined as the largest circular area encompassing the lesion on its largest axial slice. PyRadiomics (version 3.0.0; www.radiomics.io/pyradiomics.html) was utilized to propagate the ROI to corresponding multiparameter images and extract first-order radiomic features. The values of first-order features on VMI 40–200 keV and conventional images were calculated according to the methodology detailed at https://pyradiomics.readthedocs.io/en/latest/features.html#module-radiomics.firstorder. The extracted first-order features (n = 16) included: mean, maximum, minimum, median, interquartile range, entropy, uniformity, range, skewness, kurtosis, variance, 10th percentile, 90th percentile, mean absolute deviation, robust mean absolute deviation, and root mean squared.

### 2.4 Test DL model performance

All conventional images reconstructed from DLSCT were preprocessed using a bone window setting (window level: 400 HU; window width: 2000 HU). To generate the input for the DL model, two experienced radiologists **(**YC.X **and** F.X**)** manually delineated and annotated the lesion ROI on each CT slice using bounding boxes within the LabelImg software (https://pypi.org/project/labelImg/). As per the model's requirements, three consecutive CT slices were annotated per lesion: the slice demonstrating the largest lesion area and the two immediately adjacent slices. To evaluate the performance of a DL based approach, we utilized a previously developed and validated CNN model [8]. This model was originally trained and validated on a large, independent dataset of 1228 sclerotic lesions from 1008 patients, demonstrating high accuracy in differentiating BI and OBM. The model code is available at (https://github.com/Xiongyuchao/OBMORBINet).

### 2.5 Diagnostic performance comparisons

To fulfill the study's objectives, a multi-faceted approach to performance evaluation was employed. The primary analysis focused on a direct comparison between the most effective first-order radiomic feature from DLSCT and the DL model's diagnostic performance. To assess the independent contributions of spectral data and the DL model, two secondary comparisons were also performed: first-order radiomic features from VMI40–200keV images were compared with those from conventional 120-kVp images, and the performance of the DL model was compared with first-order radiomic features extracted from the same conventional images.

### 2.6 Statistical analysis

Statistical analyses were performed using SPSS software (version 22.0; IBM, NY, USA). The Kolmogorov-Smirnov test was employed to assess the normality of continuous variables. Continuous variables are presented as mean ± standard deviation or as medians with interquartile ranges (Q1, Q3) and ranges, as appropriate. The independent-samples *t*-test or Mann-Whitney *U* test was used to compare patient demographics and spectral parameters between the BI and OBM groups. Receiver operating characteristic (ROC) curve analysis was performed to evaluate the diagnostic performance of various parameters. The area under the curve (AUC), sensitivity, specificity, and 95 % confidence intervals (CI) were calculated.

AUC values were compared using the DeLong test. The optimal cutoff value for each parameter was determined using the maximum Youden index. Specificity and sensitivity values were compared using the McNemar test. A two-sided P-value < 0.05 was considered statistically significant.

## 3 Results

### 3.1 Patient characteristics

**Fig. 1:**
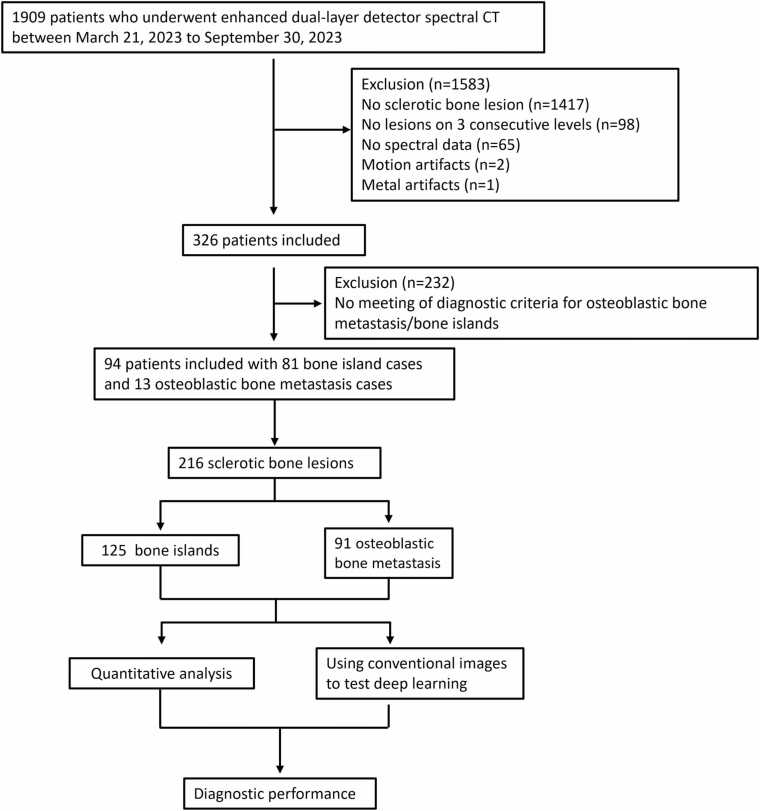
Study flowchart shows patient inclusion and exclusion criteria.

In this study, 94 patients with a total of 216 lesions were included **(**Fig. 1**)**. Among them, 81 patients (mean age 65.56 ± 12.74 years, 43 males and 38 females) had 125 BI lesions, and 13 patients (mean age 67.15 ± 6.50 years, 5 males and 8 females) had 91 OBM lesions. In the BI group, 27 patients exhibited multiple eligible bone islands: 17 with 2 BIs, 10 with 3–5 lesions, and none with more than 5 lesions. In the OBM group, 10 patients had multiple eligible lesions: 4 with 3–5 lesions, and 6 with more than 5 lesions. All patients with OBM had primary tumors in the lung (n = 10) or breast (n = 3), with 10 of these 13 patients having received prior treatment. Table 1 summarizes the clinical characteristics of the enrolled patients, including lesion counts, patient numbers, gender distribution, average age, and ROI area.

**Table 1:** Patient demographics.

Parameter	Total	BI	OBM	Statistical values	*P*
Number of lesions	216	125	91	–	–
Number of patients	94	81	13	–	–
Number of lesions per patient				8.948	.003
Isolated	57 (60.6 %)	54 (66.7 %)	3 (23.1 %)		
Multiple	37 (39.4 %)	27 (33.3 %)	10 (76.9 %)		
Sex				0.959	.381
Male	48 (51.1 %)	43 (53.1 %)	5 (38.5 %)		
Female	46 (48.9 %)	38 (46.9 %)	8 (61.5 %)		
Age (year)	65.78 ± 12.06	65.56 ± 12.74	67.15 ± 6.50	0.442	.660
ROI area (cm^2^)	38.30 ± 41.56	18.75 ± 13.53	65.16 ± 51.12	58.437	.001

BI, Bone island; OBM, osteoblastic bone metastasis; ROI, region of interest.

### 3.2 Comparison of first-order feature parameters of spectral images between BI and OBM

**Fig. 2:**
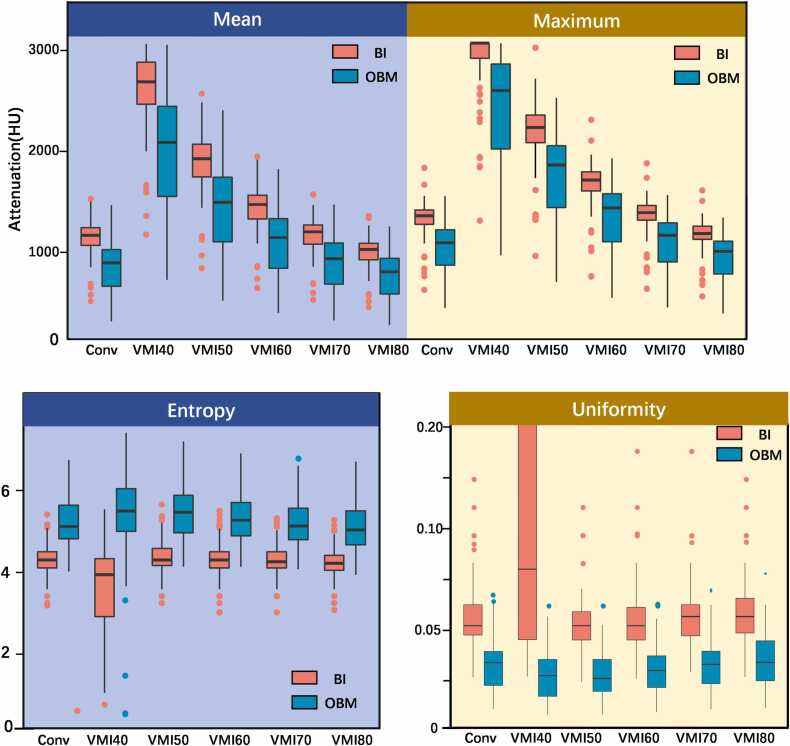
Box plots of (A) the maximum and mean, (B) entropy, and (C) uniformity of BI and OBM attenuation of conventional (Conv) and virtual monoenergetic images at 40–80 keV (VMI40–80). Dots represent individual values. Boxes indicate the upper and lower quartiles, and horizontal lines within the boxes indicate the median values. BI, Bone island; OBM, osteoblastic bone metastasis.

Table 2 and Fig. 2 present the results of the quantitative analysis for the most diagnostically relevant VMI range, with detailed results for all VMI levels (40–200 keV) provided in Supplementary Table 2. OBM demonstrated significantly lower maximum and mean attenuation values on conventional and VMI40–80keV images compared to BI (P < 0.05). Additionally, OBM exhibited significantly lower uniformity and higher entropy on conventional and VMI 40–80 keV images (all P < 0.05).

**Table 2:** Comparison of first-order feature parameters of spectral images between BI and OBM.

**Parameters**		BI	OBM	*P*
**Maximum**				
	Conventional images	1360 (1274–1419.50)	1093 (869–1230)	< .001
	VMIs			
	40 keV	3071 (2913.50–3071)	2602 (2012–2868)	< .001
	50 keV	2235 (2084.50–2363)	1863 (1432–2059)	< .001
	60 keV	1715 (1606–1799)	1437 (1103–1582)	< .001
	70 keV	1391 (1316.50–1464.50)	1165 (900–1295)	< 001
	80 keV	1185 (1127.50–1258)	1007 (783–1112)	< .001
**Mean**				
	Conventional images	1166.04 (1067.61–1244.13)	892.2 (660.71–1027.90)	< .001
	VMIs			
	40 keV	2686.62 (2466.05–2884.42)	2086.89 (1550.05–2450.84)	< .001
	50 keV	1923.96 (1744.58–2069.99)	1493.57 (1101.93–1754.87)	< .001
	60 keV	1471.24 (1326.6–1566.21)	1143.68 (840.79–1334.98)	< .001
	70 keV	1200.38 (1078.74–1270.26)	934.52 (682.27–1095)	< .001
	80 keV	1027.03 (923.52–1090.29)	804.36 (583.77–942.70)	< .001
**Uniformity**				
	Conventional images	0.05 (0.05–0.06)	0.03 (0.02–0.04)	< .001
	VMIs			
	40 keV	0.08 (0.05–0.24)	0.02 (0.02–0.03)	< .001
	50 keV	0.05 (0.05–0.06)	0.03 (0.02–0.04)	< .001
	60 keV	0.05 (0.05–0.06)	0.03 (0.02–0.04)	< .001
	70 keV	0.06 (0.05–0.06)	0.03 (0.02–0.04)	< .001
	80 keV	0.06 ± 0.02	0.03 ± 0.01	< .001
**Entropy**				
	Conventional images	4.30 (4.11–4.50)	5.12 (4.8–5.65)	< .001
	VMIs			
	40 keV	3.94 (2.92–4.36)	5.49 (4.99–6.05)	< .001
	50 keV	4.30 (4.17–4.58)	5.46 (4.95–5.88)	< .001
	60 keV	4.30 (4.11–4.50)	5.27 (4.89–5.72)	< .001
	70 keV	4.25 (4.11–4.50)	5.13 (4.79–5.61)	< .001
	80 keV	4.22 (4.04–4.42)	5.03 (4.66–5.53)	< .001

BI, bone island; OBM, osteoblastic bone metastasis; VMI, virtual monoenergetic image.

### 3.3 Diagnostic performance of spectral images and DL model

**Fig. 3:**
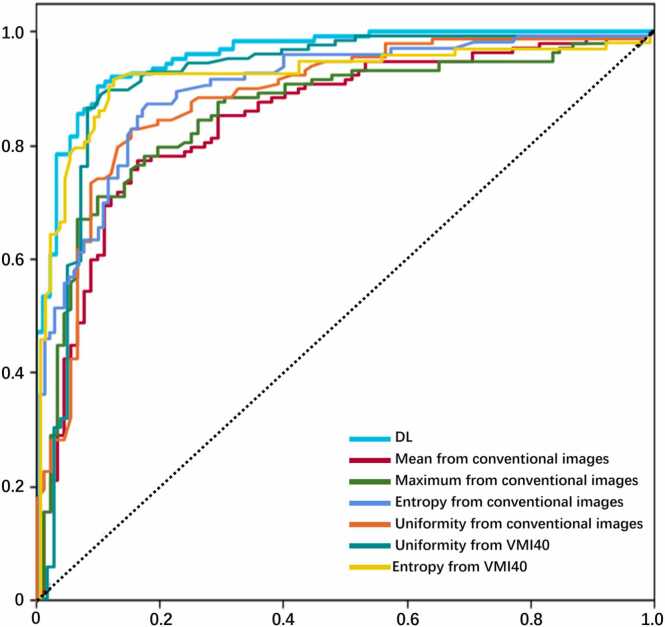
Receiver-operating characteristic curves of each parameter and DL model. DL, Deep learning.

**Fig. 4:**
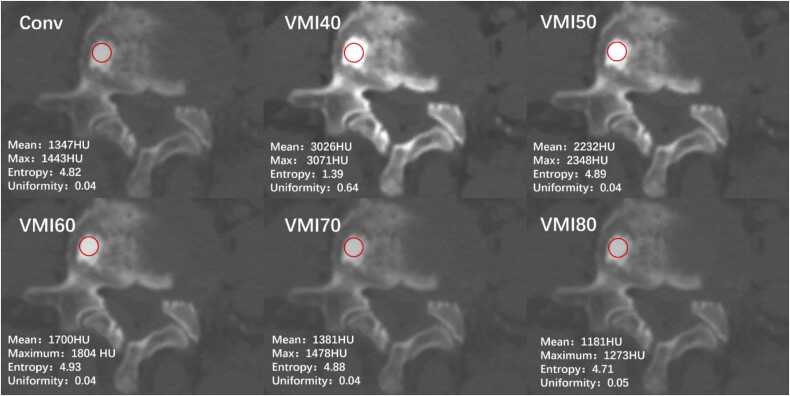
Axial CT images in an 87-year-old female patient with BI lesion. Maximum, minimum, entropy, and uniformity of lesions measured on conventional images and VMI40–80. BI, Bone island; CT, computed tomography.

**Fig. 5:**
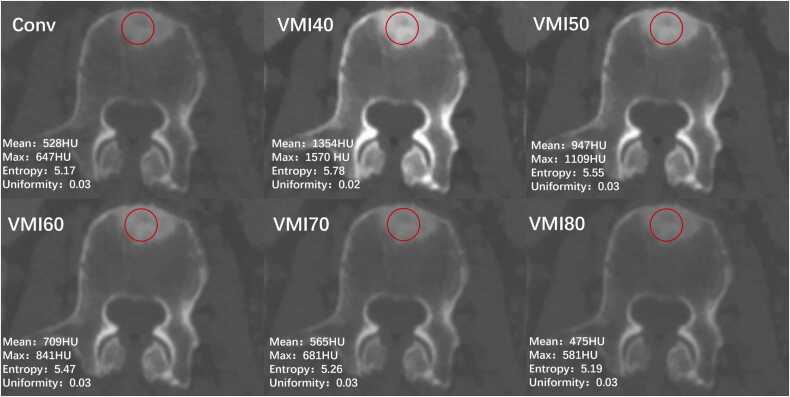
Axial CT images in a 71-year-old male patient with OBM from gastric cancer. Maximum, minimum, entropy, and uniformity of lesions measured on conventional and VMI40–80 images. CT, Computed tomography; OBM, osteoblastic bone metastasis.

Table 3 and Fig. 3 present the results of the ROC analysis, and detailed results are available in Supplementary Table 3. Among the mean values of all individual spectral parameters, conventional images exhibited the highest diagnostic performance in differentiating OBM and BI, with a cutoff value of 1050.7, an AUC of 0.86, a sensitivity of 78 %, and a specificity of 84 %. Considering the maximum values of all individual spectral parameters, conventional images again demonstrated the highest performance, with a cutoff value of 1301, an AUC of 0.87, a sensitivity of 72 %, and a specificity of 90 %. For uniformity, VM40keV images showed the highest performance (cutoff: 0.042; AUC: 0.93; sensitivity: 90 %; specificity: 90 %). Similarly, for entropy, VM40keV images exhibited the highest performance (cutoff: 4.593; AUC: 0.93; sensitivity: 92 %; specificity: 88 %). Consequently, the entropy and uniformity of VM40keV images, along with the mean and maximum values from conventional images, were selected for subsequent analyses. Representative cases are illustrated in Fig. 4, Fig. 5.

**Table 3:** Diagnostic performance of first-order feature parameters of spectral images in differentiating between BI and OBM.

**Parameters**		Cutoff	AUC (95 % CI)	Sensitivity (95 % CI)	Specificity (95 % CI)
**Maximum**					
	Conventional images	1301	0.86 [0.81, 0.91]	72 (66/91) [64,79]	90 (113/125) [84,96]
	VMIs				
	40 keV	2893	0.83 [0.78, 0.89]	79 (72/91) [72,86]	81 (102/125) [73,89]
	50 keV	2074	0.84 [0.78, 0.89]	80 (73/91) [73,87]	79 (99/125) [70,87]
	60 keV	1604	0.84 [0.78, 0.89]	76 (70/91) [68,83]	81 (102/125) [73,89]
	70 keV	1310	0.83 [0.78, 0.89]	76 (69/91) [68,83]	79 (99/125) [70,87]
	80 keV	1136	0.83 [0.78, 0.88]	74 (68/91) [66,82]	81 (102/125) [73,89]
**Mean**					
	Conventional images	1051	0.85 [0.80, 0.90]	78 (71/91) [71,85]	83 (104/125) [75,91]
	VMIs				
	40 keV	2454	0.83 [0.77, 0.89]	78 (71/91) [71,85]	78 (98/125) [69,86]
	50 keV	1796	0.83 [0.77, 0.89]	72 (66/91) [65,80]	83 (104/125) [75,91]
	60 keV	1365	0.83 [0.77, 0.88]	73 (67/91) [65,81]	81 (102/125) [73,89]
	70 keV	1148	0.82 [0.76, 0.88]	64 (58/91) [55,72]	89 (111/125) [82,95]
	80 keV	975	0.82 [0.76, 0.87]	64 (58/91) [55,72]	87 (110/125) [81,94]
**Uniformity**					
	Conventional images	0.04	0.89 [0.84, 0.93]	84 (76/91) [77,90]	84 (106/125) [77,92]
	VMIs				
	40 keV	0.04	0.93 [0.89, 0.97]	89 (82/91) [84,95]	90 (113/125) [84,96]
	50 keV	0.04	0.90 [0.86, 0.94]	80 (73/91) [73,87]	86 (109/125) [79,93]
	60 keV	0.04	0.90 [0.86, 0.94]	81 (74/91) [74,88]	86 (109/125) [79,93]
	70 keV	0.04	0.88 [0.84, 0.93]	82 (75/91) [75,89]	81 (102/125) [73,89]
	80 keV	0.05	0.89 [0.85, 0.93]	80 (72.8/91) [73,87]	86 (109/125) [79,93]
**Entropy**					
	Conventional images	4.63	0.91 [0.87, 0.94]	87 (79.989/91) [81,94]	82 (103/125) [75,89]
	VMIs				
	40 keV	4.59	0.93 [0.89, 0.97]	92 (84/91) [86,97]	88 (110/125) [82,93]
	50 keV	4.59	0.91 [0.88, 0.95]	93 (85/91) [88,98]	77 (97/125) [70,84]
	60 keV	4.64	0.91 [0.88, 0.95]	90 (82/91) [84,96]	80 (101/125) [73,87]
	70 keV	4.59	0.90 [0.86, 0.94]	89 (81/91) [82,95]	80 (100/125) [73,87]
	80 keV	4.49	0.91 [0.87, 0.94]	91 (83/91) [85,97]	80 (100/125) [73,87]

AUC, Area under the curve; BI, bone island; CI, confidence interval; OBM, osteoblastic bone metastasis; VMI, virtual monoenergetic image.

The DL model, utilizing conventional images, achieved an AUC of 0.96, a sensitivity of 62 %, and a specificity of 96 %.

### 3.4 Comparison of diagnostic performance between spectral images and the DL model

The DeLong test revealed no statistically significant difference between the highest AUC values of DLSCT (entropy from VMI40keV images) and the DL model [0.93 (95 % CI: 0.90–0.97) vs. 0.96 (95 % CI: 0.96–0.96); *P* = 0.274]. Both of these AUC values were significantly higher than those obtained for the mean [0.86 (95 % CI: 0.81–0.91); *P* < 0.05 for both] and maximum [0.87 (95 % CI: 0.82–0.92); *P* < 0.05 for both] values from conventional images. The DL model demonstrated significantly improved discrimination compared to all first-order feature parameters derived from conventional images (*P* < 0.05).

The entropy and uniformity of VMI40keV images exhibited significantly greater AUC values than the mean and maximum values of conventional images (*P* < 0.05). However, the entropy and uniformity of VMI40keV images showed comparable AUC values to the entropy and uniformity of conventional images (P > 0.05).

The sensitivity of entropy from VMI40keV images was significantly higher than that of the DL model (92 % [84 of 91 OBM lesions] vs. 62 % [56 of 91 OBM lesions]; *P* = 0.002). However, the specificity was not significantly different (88 % [110 of 125 BI lesions] vs. 96 % [123 of 125 BI lesions]; *P* = 0.07).

## 4 Discussion

DLSCT offers the advantage of acquiring spectral data in a single exposure, obviating the need for specialized scanning modes or compromises in spectral scanning protocols [19], [20], [21]. The resulting spectral data can be transformed into valuable diagnostic information and seamlessly integrated into the radiologist's workflow [22]. DLSCT enables the characterization of osteogenic lesions exceeding typical Hounsfield unit thresholds through spectral-based imaging [22]. The clinical application of spectral CT has demonstrated the potential to enhance both diagnostic accuracy and efficiency [9], [13], [23]. Similarly, DL models have shown promise in various clinical applications, including the classification of benign tumors and lesions [24], [25], gene expression profiling [26], and prognostic assessment [27], [28]. DL models also offer the potential to improve diagnostic efficiency and reduce radiologist workload [29], [30]. Therefore, both DLSCT and DL hold the potential for providing point-of-care, high-accuracy differentiation between BI and OBM. However, a direct comparison of their diagnostic performance in distinguishing between BI and OBM lesions has been lacking. This study addresses this gap by providing a novel comparison of DLSCT, a DL model, and conventional CT in differentiating BI from OBM.

The present study demonstrated that DLSCT exhibited comparable diagnostic performance to the DL model (AUC: 0.93 vs. 0.96; *P* = 0.274) in distinguishing between BI and OBM, and superior performance compared to conventional CT based on Hounsfield unit measurements (highest AUC: 0.93 vs. 0.87; *P* < 0.05). Notably, the sensitivity of entropy derived from VMI40keV images reconstructed from DLSCT was significantly higher than that of both the DL model and conventional CT images in the diagnosis of OBM (sensitivity: 92 %, 62 %, and 88 %, respectively; P < 0.05 for all comparisons).

A key advantage of DLSCT is its ability to retrospectively evaluate all clinical cases after a CT scan, without requiring additional radiation dose or scan time [31]. However, the analysis and interpretation of the large volume of quantitative parameters generated by DLSCT can be challenging for radiologists. Thus, there is a pressing need for readily accessible reference data for spectral parameters associated with specific diseases, obtainable through simple image delineation. Based on single-slice image delineation, the present study determined that the entropy of VMI40keV images derived from DLSCT possessed the highest diagnostic value. Its performance in differentiating BI and OBM was comparable to that of the DL model and superior to that of conventional CT based on attenuation measurements.

Entropy, a key first-order radiomic feature, serves as a numerical descriptor of a lesion's texture, quantifying the randomness and heterogeneity of its internal pixel intensity distribution[32]. A lesion with a high entropy value is characterized by a more heterogeneous, varied pixel pattern, while a lesion with low entropy appears more uniform. This metric holds significant physiological relevance in the context of bone lesions. Bone islands are typically uniform sclerotic foci, resulting in low entropy values. In contrast, osteoblastic metastases are inherently more heterogeneous due to a combination of disorganized bone formation, tumor cells, and areas of necrosis, leading to higher entropy values. These findings align with previous studies[33], [34] that have correlated CT feature entropy with histology and bone quality, suggesting its utility as a quantitative indicator for assessing the internal architecture of bone lesions. As multi-center studies expand, integrating entropy values into post-processing systems could streamline the DLSCT-based diagnosis of BI and OBM.

Our finding that the entropy of VMI40keV images from DLSCT demonstrated a significantly higher sensitivity (92 %) in diagnosing OBM, compared to both the DL model and conventional CT, is particularly valuable given the inclusion of post-treatment OBMs in our cohort. As noted, osteoblastic repair reactions from treatments like chemotherapy and bisphosphonates can increase the density of OBMs[3], [4], [5], [6], which can reduce the sensitivity of diagnostic methods that rely on simple attenuation measurements. Our study demonstrates that DLSCT, by utilizing spectral information, provides a more robust diagnostic solution for these difficult cases. This superior performance is crucial for real-world scenarios where a patient's complete treatment history may be unavailable or unknown, and there is a risk of misclassifying a treated OBM as a benign bone island. Therefore, the high sensitivity of DLSCT in this challenging subgroup has the potential to reduce the likelihood of missed diagnoses and inaccurate tumor staging. The observed large variability in the uniformity of BIs on VMI40 keV images can be attributed to the energy-dependent properties of the spectral images. Low-energy images, such as VMI40 keV, are more sensitive to variations in material composition due to the photoelectric effect[35]. While most BIs are homogeneous, some may contain minute, non-uniform features such as small fibrous components or micro-vascular channels that are not visible on conventional images[11]. The VMI40 keV images magnify these subtle differences, leading to a wider range of uniformity values for BIs. This finding highlights the ability of DLSCT to reveal underlying tissue heterogeneity that is not apparent on standard images and provides a potential explanation for the superior diagnostic performance of VMI40 keV image-derived entropy in our study.

Regarding the application of DL models in distinguishing between BI and OBM, the previously developed DL model by the author demonstrated excellent performance in both internal and external validation sets [8]. This DL model continued to perform well on conventional images generated from DLSCT. However, no significant difference was observed between the DL model in this study and DLSCT in differentiating BI and OBM. This may be attributed to the differing strengths of DL and DLSCT in diagnosing BI and OBM. The DL model's strength lies in its ability to extract information, including, as previously mentioned, the boundaries of BI and OBM, whereas spectral CT is limited to measuring multiparameter spectral values within the lesion and cannot extract boundary information. BI is characterized by distinctive radiating bone striations ("thorn rays") that create feathery or brush-like borders with the trabecular bone [36], while OBM can exhibit destruction of the adjacent cortical bone. Another advantage of the DL model is its reliance on three consecutive CT image slices as input. This allows the DL model to extract certain three-dimensional features of the lesions, whereas spectral CT measurement values do not capture this three-dimensional information. Conversely, DLSCT offers the advantage of accurately quantifying first-order parameters of lesions. In contrast, the DL model relies on identifying image features and classifying lesions within a specific window width and level, potentially obscuring certain parameter values that could be beneficial in differentiating BI and OBM [29], [30].

While DL and DLSCT demonstrated comparable performance in differentiating BI and OBM, their applicability in various clinical settings may differ. The cost of implementing DL is substantially lower than acquiring a new DLSCT system [37]. Therefore, DL may be a more suitable option for assisting in the diagnosis of osteogenic lesions in resource-constrained or economically underdeveloped healthcare settings. For organizations that already have DLSCT capabilities, utilizing DLSCT for diagnosing osteogenic lesions may be preferable, as DLSCT parameters are more readily interpretable than those of DL, which is often considered a "black box" with a decision-making process that is difficult to understand and interpret [38]. Furthermore, DLSCT parameters are readily available and well-integrated into the clinical workflow. While our study compared DLSCT and a DL model as independent approaches, a promising avenue for future research is to explore the optimal parameter map from DLSCT in combination with DL techniques. This hybrid approach could potentially leverage the strengths of both methods to further improve diagnostic accuracy and is a clear direction for our future work. The authors posit that future research exploring the optimal parameter map from DLSCT for distinguishing between lesions, combined with DL techniques, represents a promising avenue for improving disease diagnosis.

This study has several limitations. First, as a single-center retrospective study with a relatively small sample size, it is subject to inherent selection bias. Therefore, the findings require validation through larger, multi-center studies. Second, the results were obtained using images from detector-based dual-energy CT. Consequently, the optimal energy levels and diagnostic performance may not be directly extrapolated to source-based dual-energy CT systems. Third, lesion diagnosis was based on follow-up results rather than histopathological examination, a common limitation in studies of osteogenic lesions [6].

In conclusion, Radiomic features from DLSCT demonstrated utility in distinguishing between BI and OBM, with diagnostic performance comparable to that of a DL model. Furthermore, VMI40keV images generated from DLSCT enabled the differentiation of OBM from BI with significantly higher sensitivity compared to both the DL model and conventional CT. This enhanced sensitivity has the potential to reduce the likelihood of incorrect tumor staging and missed diagnoses, particularly in patients with OBM where the tumor history or treatment history is unknown.

## Author statement

All authors have confirmed that each author have seen and approved the final version of the manuscript being submitted. All authors warrant that the article is the authors' original work, hasn't received prior publication and isn't under consideration for publication elsewhere.

## CRediT authorship contribution statement

**Fan Xu:** Writing – review & editing, Writing – original draft, Software, Resources, Methodology, Formal analysis, Data curation, Conceptualization. **Li Wu:** Writing – review & editing, Writing – original draft, Supervision, Software, Methodology, Investigation, Data curation, Conceptualization. **Jiahui Ou:** Writing – review & editing, Writing – original draft, Visualization, Supervision, Software, Methodology, Formal analysis, Data curation, Conceptualization. **Yuchao Xiong:** Writing – review & editing, Writing – original draft, Validation, Supervision, Software, Methodology, Formal analysis, Data curation, Conceptualization. **Wei Guo:** Writing – review & editing, Writing – original draft, Visualization, Methodology, Funding acquisition, Formal analysis, Data curation, Conceptualization. **Xuwen Zeng:** Writing – review & editing, Writing – original draft, Visualization, Validation, Supervision, Methodology, Formal analysis, Data curation, Conceptualization.

## Ethics approval

The study was approved by the Ethics Committee of the Guangzhou Red Cross Hospital (2023–262–01), and confirm that informed consent was obtained from all participants and/or their legal guardians. The study was conducted in accordance with the Declaration of Helsinki.

## Declaration of Generative AI and AI-assisted technologies in the writing process

During the preparation of this work, the author used Gemini to polish the text of the article. After using this tool, the author reviewed and edited the content as needed and takes full responsibility for the content of the published article.

## Funding

This study has received funding by Knowledge Innovation Project of Wuhan, China [grant numbers: 2023020201020544] (W.G.).

## Declaration of Competing Interest

The authors declare that they have no known competing financial interests or personal relationships that could have appeared to influence the work reported in this paper.

## Data Availability

The original contributions presented in the study are included in the article. Further inquiries can be directed to the corresponding author.

## Footnotes

Appendix A Supplementary data associated with this article can be found in the online version at doi:10.1016/j.ejro.2025.100679.
