# Supplementary material for: Diagnostic performance of dual-layer spectral CT Radiomics and deep learning for differentiating osteoblastic bone metastases from bone islands
Source: Eur J Radiol Open. 2025 Aug 20;15:100679. doi: 10.1016/j.ejro.2025.100679 (PMC12396480; doi:10.1016/j.ejro.2025.100679)
Supplement: Supplementary file 1 — Supplementary material [file mmc1.doc]

**Supplemental Material**

Supplementary table 1. CT protocols of different examination sites

| Parameters | Chest | Abdomen | Adrenal gland | Thyroid |
| --- | --- | --- | --- | --- |
| Tube voltage | 120 kVp | 120 kVp | 120 kVp | 120 kVp |
| Tube current | 50-350 mAs with automatic tube current modulation | 50-350 mAs with automatic tube current modulation | 50-350 mAs with automatic tube current modulation | 50-350 mAs with automatic tube current modulation |
| Automated tube current modulation | Care Dose | Care Dose | Care Dose | Care Dose |
| Rotation time | 0.27 s | 0.33 s | 0.33 s | 0.40 s |
| Collimation | Auto | Auto | Auto | Auto |
| Slice thickness | 3 mm | 3 mm | 3 mm | 3 mm |
| Slice interva | 1.5 mm | 1.5 mm | 1.5 mm | 1.5 mm |
| Acquired plane | Axial | Axial | Axial | Axial |
| Contrast agent type | Iopromide, Ultravist 300; Bayer Healthcare, Berlin, Germany | Iopromide, Ultravist 300; Bayer Healthcare, Berlin, Germany | Iopromide, Ultravist 300; Bayer Healthcare, Berlin, Germany | Iopromide, Ultravist 300; Bayer Healthcare, Berlin, Germany |
| Contrast agent dosage | Based on body weight: 80-120 mL | Based on body weight: 80-120 mL | Based on body weight: 80-120 mL | Based on body weight: 80-120 mL |
| Contrast agent infused rate | 2 mL/s | 2 mL/s | 2 mL/s | 2 mL/s |
| Reconstruction algorithm | iDose 4, level 3 | iDose 4, level 3 | iDose 4, level 3 | iDose 4, level 3 |
| Reconstruction kernel | Standard(B) | Standard(B) | Standard(B) | Standard(B) |

Supplementary table 2. Comparison of first-order feature parameters of spectral images between BI and OBM

| **Parameters** | | BI | OBM | *P* |
| --- | --- | --- | --- | --- |
| **Entropy** | |  |  |  |
|  | Conventional images | 4.3(4.11-4.5) | 5.12(4.8-5.65) | 0 |
|  | VMIs |  |  |  |
|  | 40 keV | 3.94(2.92-4.36) | 5.49(4.99-6.05) | 0 |
|  | 50 keV | 4.3(4.17-4.58) | 5.46(4.95-5.88) | 0 |
|  | 60 keV | 4.3(4.11-4.5) | 5.27(4.89-5.72) | 0 |
|  | 70 keV | 4.25(4.11-4.5) | 5.13(4.79-5.61) | 0 |
|  | 80 keV | 4.22(4.04-4.42) | 5.03(4.66-5.53) | 0 |
|  | 90 keV | 4.22(4.05-4.42) | 5.02(4.69-5.38) | 0 |
|  | 100 keV | 4.2(4.01-4.42) | 4.96(4.67-5.34) | 0 |
|  | 110 keV | 4.2(4.08-4.39) | 4.89(4.54-5.37) | 0 |
|  | 120 keV | 4.2(4.02-4.42) | 4.89(4.59-5.35) | 0 |
|  | 130 keV | 4.2(4.05-4.39) | 4.88(4.57-5.32) | 0 |
|  | 140 keV | 4.17(4.01-4.36) | 4.8(4.47-5.27) | 0 |
|  | 150 keV | 4.2(4.01-4.39) | 4.84(4.52-5.26) | 0 |
|  | 160 keV | 4.16(4-4.38) | 4.85(4.54-5.26) | 0 |
|  | 170 keV | 4.15(4.01-4.33) | 4.79(4.54-5.24) | 0 |
|  | 180 keV | 4.17(3.97-4.33) | 4.77(4.48-5.27) | 0 |
|  | 190 keV | 4.17(3.94-4.33) | 4.76(4.49-5.26) | 0 |
|  | 200 keV | 4.11(3.94-4.33) | 4.74(4.52-5.23) | 0 |
| **Minimum** | |  |  |  |
|  | Conventional images | 818(681.5-976.5) | 608(359-811) | 0 |
|  | VMIs |  |  |  |
|  | 40 keV | 1902(1621-2310) | 1445(934-1985) | 0 |
|  | 50 keV | 1348(1149.5-1630) | 1014(638-1408) | 0 |
|  | 60 keV | 1018(875.5-1231) | 769(477-1068) | 0 |
|  | 70 keV | 829(699-988.5) | 621(383-864) | 0 |
|  | 80 keV | 707(596-842.5) | 529(316-733) | 0 |
|  | 90 keV | 627(521-748.5) | 469(286-642) | 0 |
|  | 100 keV | 570(474.5-686) | 428(260-582) | 0 |
|  | 110 keV | 532(439-640) | 400(242-543) | 0 |
|  | 120 keV | 500(418-602) | 380(228-515) | 0 |
|  | 130 keV | 487.23±143.02 | 358.41±156.13 | 0 |
|  | 140 keV | 470.91±139 | 345.66±151.32 | 0 |
|  | 150 keV | 458.18±135.92 | 335.67±147.53 | 0 |
|  | 160 keV | 448.1±133.46 | 327.8±144.54 | 0 |
|  | 170 keV | 439.91±131.53 | 321.45±142.17 | 0 |
|  | 180 keV | 433.28±129.9 | 316.22±140.26 | 0 |
|  | 190 keV | 427.75±128.68 | 311.99±138.61 | 0 |
|  | 200 keV | 423.1±127.46 | 308.33±137.31 | 0 |
| **Maximum** | |  |  |  |
|  | Conventional images | 1360(1274-1419.5) | 1093(869-1230) | 0 |
|  | VMIs |  |  |  |
|  | 40 keV | 3071(2913.5-3071) | 2602(2012-2868) | 0 |
|  | 50 keV | 2235(2084.5-2363) | 1863(1432-2059) | 0 |
|  | 60 keV | 1715(1606-1799) | 1437(1103-1582) | 0 |
|  | 70 keV | 1391(1316.5-1464.5) | 1165(900-1295) | 0 |
|  | 80 keV | 1185(1127.5-1258) | 1007(783-1112) | 0 |
|  | 90 keV | 1059(1001.5-1125) | 907(699-997) | 0 |
|  | 100 keV | 967(916-1032.5) | 833(643-916) | 0 |
|  | 110 keV | 908(850-965) | 777(602-855) | 0 |
|  | 120 keV | 865(805-922) | 735(571-816) | 0 |
|  | 130 keV | 831(773-891) | 706(551-786) | 0 |
|  | 140 keV | 805(749-865) | 687(535-760) | 0 |
|  | 150 keV | 785(730-845) | 674(522-742) | 0 |
|  | 160 keV | 769(715-829) | 664(513-728) | 0 |
|  | 170 keV | 755(702-818) | 656(505-717) | 0 |
|  | 180 keV | 744(691-807) | 649(499-708) | 0 |
|  | 190 keV | 736(682-798) | 643(493-700) | 0 |
|  | 200 keV | 729(674-791) | 639(489-694) | 0 |
| **10^th^ percentile** | |  |  |  |
|  | Conventional images | 982.3(833.35-1093.15) | 737(525-907.4) | 0 |
|  | VMIs |  |  |  |
|  | 40 keV | 2285.7(1932-2595.25) | 1752.5(1191.1-2207) | 0 |
|  | 50 keV | 1615(1364.3-1830.55) | 1265.3(856.7-1575.5) | 0 |
|  | 60 keV | 1227.6(1040.15-1387.7) | 952.4(660.5-1200.5) | 0 |
|  | 70 keV | 1003.5(841.4-1110.95) | 782.8(543-966.5) | 0 |
|  | 80 keV | 848.6(714.45-941.05) | 677(467.8-819.4) | 0 |
|  | 90 keV | 753(634.6-839.55) | 601.6(406.5-733) | 0 |
|  | 100 keV | 688(578.9-771.55) | 549.4(372.5-671) | 0 |
|  | 110 keV | 636.4(537.5-722.2) | 513.3(348.6-627) | 0 |
|  | 120 keV | 601.3(509.3-678.4) | 486.2(330.8-596) | 0 |
|  | 130 keV | 574.2(487.6-650.9) | 466.1(317.9-572) | 0 |
|  | 140 keV | 554(471-631.9) | 451.2(308-554) | 0 |
|  | 150 keV | 539(458.1-617.9) | 440(301-540) | 0 |
|  | 160 keV | 526.9(448.1-606.6) | 430.1(295.9-529) | 0 |
|  | 170 keV | 516.9(439.2-597.6) | 423.1(291.8-520) | 0 |
|  | 180 keV | 508.8(432.2-589.9) | 416.1(288.8-513) | 0 |
|  | 190 keV | 502.1(426.3-583.6) | 411.1(285-507) | 0 |
|  | 200 keV | 496.4(421.3-578.6) | 407.1(282-502) | 0 |
| **90^th^ percentile** | |  |  |  |
|  | Conventional images | 1323.7(1227.4-1396.3) | 1001(803-1143.8) | 0 |
|  | VMIs |  |  |  |
|  | 40 keV | 3060.2(2865.65-3071) | 2371.5(1856.4-2723) | 0 |
|  | 50 keV | 2194(2045.95-2326.2) | 1692(1330.9-1943) | 0 |
|  | 60 keV | 1671.7(1545.65-1771) | 1303.2(1018.6-1485) | 0 |
|  | 70 keV | 1357(1258.5-1436.25) | 1066.4(836.8-1212) | 0 |
|  | 80 keV | 1164(1078.7-1233.05) | 916(714-1041) | 0 |
|  | 90 keV | 1035(959.8-1098) | 822.2(635.3-930) | 0 |
|  | 100 keV | 942.4(877.5-1005.95) | 758(584.2-854.2) | 0 |
|  | 110 keV | 883.4(816.7-941.9) | 712.2(548-802.1) | 0 |
|  | 120 keV | 840.4(776-896) | 678.6(522.8-764.7) | 0 |
|  | 130 keV | 807(747.7-863) | 653(502.7-736.8) | 0 |
|  | 140 keV | 779.8(724.7-836.4) | 632(486.8-712.8) | 0 |
|  | 150 keV | 761(706.7-817.8) | 616(474-696) | 0 |
|  | 160 keV | 744.7(690.4-802) | 604.4(464.4-681.8) | 0 |
|  | 170 keV | 731.7(676.4-789.2) | 595(456.4-670.8) | 0 |
|  | 180 keV | 721(665.3-779.8) | 588(450.4-660.8) | 0 |
|  | 190 keV | 713(655.4-771.8) | 581.6(444.4-652.8) | 0 |
|  | 200 keV | 707(648.3-764.4) | 576.6(440.4-645.8) | 0 |
| **Mean** | |  |  |  |
|  | Conventional images | 1166.04(1067.61-1244.13) | 892.2(660.71-1027.9) | 0 |
|  | VMIs |  |  |  |
|  | 40 keV | 2686.62(2466.05-2884.42) | 2086.89(1550.05-2450.84) | 0 |
|  | 50 keV | 1923.96(1744.58-2069.99) | 1493.57(1101.93-1754.87) | 0 |
|  | 60 keV | 1471.24(1326.6-1566.21) | 1143.68(840.79-1334.98) | 0 |
|  | 70 keV | 1200.38(1078.74-1270.26) | 934.52(682.27-1095) | 0 |
|  | 80 keV | 1027.03(923.52-1090.29) | 804.36(583.77-942.7) | 0 |
|  | 90 keV | 909.92(815.84-966.52) | 719.37(519.39-837.25) | 0 |
|  | 100 keV | 830.24(740.18-888.05) | 661.64(475.6-768.14) | 0 |
|  | 110 keV | 774.19(691.14-831.73) | 621.02(444.81-721.32) | 0 |
|  | 120 keV | 734(655.33-790) | 590.81(422.48-685.82) | 0 |
|  | 130 keV | 702.27(626.03-762.54) | 566.97(405.87-657.8) | 0 |
|  | 140 keV | 679.29(606.05-740.58) | 548.57(393.09-637.64) | 0 |
|  | 150 keV | 661.92(590.57-722.25) | 534.45(383.13-621.91) | 0 |
|  | 160 keV | 648.17(578.16-709.38) | 523.93(375.26-609.43) | 0 |
|  | 170 keV | 637.17(568.3-696.25) | 515.45(368.94-599.39) | 0 |
|  | 180 keV | 626.81(560.16-686.63) | 508.64(363.74-591.14) | 0 |
|  | 190 keV | 617.73(553.46-678.08) | 502.93(359.41-584.24) | 0 |
|  | 200 keV | 610.3(547.86-671.46) | 498.11(355.84-578.24) | 0 |
| **Median** | |  |  |  |
|  | Conventional images | 1205(1097.5-1293.25) | 894(674-1038.5) | 0 |
|  | VMIs |  |  |  |
|  | 40 keV | 2799(2581-3021.5) | 2103.5(1581-2470) | 0 |
|  | 50 keV | 1990.5(1829-2138.25) | 1512(1120-1764) | 0 |
|  | 60 keV | 1507(1386-1636) | 1156(849-1354) | 0 |
|  | 70 keV | 1222(1118.5-1331.25) | 943(687-1108) | 0 |
|  | 80 keV | 1043(954-1136.5) | 809(589-952) | 0 |
|  | 90 keV | 925(839.5-1006.5) | 723(526.5-847) | 0 |
|  | 100 keV | 847.5(760-919) | 662(481-775) | 0 |
|  | 110 keV | 793(706-862) | 622(449-726) | 0 |
|  | 120 keV | 750.5(667-824) | 593.5(426-691) | 0 |
|  | 130 keV | 719(639-790) | 573(409-666) | 0 |
|  | 140 keV | 694.5(618-764) | 555(396-645) | 0 |
|  | 150 keV | 676(600-744) | 542(385-629) | 0 |
|  | 160 keV | 661(589-728) | 531.5(377-616) | 0 |
|  | 170 keV | 648(580-715) | 523(370.5-606) | 0 |
|  | 180 keV | 638(573-706) | 516.5(364.5-598) | 0 |
|  | 190 keV | 630(566-700.5) | 510.5(360.5-592) | 0 |
|  | 200 keV | 623.5(560-693) | 505.5(356.5-586) | 0 |
| **Interquartile range** | |  |  |  |
|  | Conventional images | 182.5(112-262) | 116(96.5-168) | 0 |
|  | VMIs |  |  |  |
|  | 40 keV | 343(194.38-579) | 272.25(211-381) | 0.022 |
|  | 50 keV | 303.25(174.38-455.88) | 200(155.25-267) | 0 |
|  | 60 keV | 240.75(138.75-341.63) | 154.5(119.25-214) | 0 |
|  | 70 keV | 199(118.38-274.13) | 123.5(103-171) | 0 |
|  | 80 keV | 176.5(107.88-234.88) | 106(86-152) | 0 |
|  | 90 keV | 151.75(99.38-215.75) | 97(78-136.25) | 0 |
|  | 100 keV | 142.75(92.38-205.25) | 87.5(71.75-125.25) | 0 |
|  | 110 keV | 136.5(84.75-195.25) | 83(68-116.25) | 0 |
|  | 120 keV | 131(83.25-186.5) | 80(65-113) | 0 |
|  | 130 keV | 127.75(81-180) | 78(64-111) | 0 |
|  | 140 keV | 124(80-174) | 76(62-108) | 0 |
|  | 150 keV | 121.5(79-169.25) | 75(60-105) | 0 |
|  | 160 keV | 120.5(79-166.25) | 73(59.5-103) | 0 |
|  | 170 keV | 118.25(78.25-164) | 73(58-102) | 0 |
|  | 180 keV | 116(77-162) | 73(58-101) | 0 |
|  | 190 keV | 115(76.5-160) | 73(57-100.75) | 0 |
|  | 200 keV | 113(74.75-159.5) | 73(57-100.75) | 0 |
| **Range** | |  |  |  |
|  | Conventional images | 500(337.5-651) | 414(314-521) | 0.016 |
|  | VMIs |  |  |  |
|  | 40 keV | 994.95±462.72 | 963.03±426.7 | 0.606 |
|  | 50 keV | 794(555-1062) | 694(482-834) | 0.014 |
|  | 60 keV | 624(424.5-823) | 533(374-661) | 0.012 |
|  | 70 keV | 523(344.5-668) | 446(315-545) | 0.010 |
|  | 80 keV | 458.02±171.92 | 405.91±151.71 | 0.022 |
|  | 90 keV | 413.43±151.93 | 369.88±136.04 | 0.031 |
|  | 100 keV | 383.34±138.47 | 345.87±125.48 | 0.042 |
|  | 110 keV | 361.94±130.38 | 329.13±118.14 | 0.060 |
|  | 120 keV | 346.62±123.67 | 317.24±113 | 0.076 |
|  | 130 keV | 335.26±118.83 | 308.45±109.19 | 0.093 |
|  | 140 keV | 326.61±115.08 | 301.66±106.32 | 0.107 |
|  | 150 keV | 319.85±112.24 | 296.45±104.13 | 0.122 |
|  | 160 keV | 314.5±109.96 | 292.31±102.46 | 0.134 |
|  | 170 keV | 310.24±108.08 | 288.99±101.08 | 0.145 |
|  | 180 keV | 306.71±106.64 | 286.32±99.95 | 0.157 |
|  | 190 keV | 303.91±105.44 | 283.95±99 | 0.161 |
|  | 200 keV | 301.43±104.4 | 282.24±98.23 | 0.174 |
| **Mean absolute deviation** | |  |  |  |
|  | Conventional images | 113.07(72.85-148.29) | 70.63(58.97-98.05) | 0 |
|  | VMIs |  |  |  |
|  | 40 keV | 231.33(133.24-329.93) | 168.67(127.07-222.3) | 0 |
|  | 50 keV | 189.86(115.42-253.87) | 122.93(95.59-163.62) | 0 |
|  | 60 keV | 149.11(90.57-193.94) | 94.39(74.05-121.95) | 0 |
|  | 70 keV | 123.43(76.35-156.95) | 77.72(60.85-103.24) | 0 |
|  | 80 keV | 106.69(69.84-135.56) | 67.6(53.36-87.69) | 0 |
|  | 90 keV | 95.77(65.59-122.77) | 60.61(48.75-80.44) | 0 |
|  | 100 keV | 86.65(62.15-113.58) | 56.56(45.65-76.1) | 0 |
|  | 110 keV | 82.96(58.78-107.24) | 53.29(43.89-72.39) | 0 |
|  | 120 keV | 80.25(56.17-103.69) | 51.28(42.12-68.96) | 0 |
|  | 130 keV | 77.06(54.28-100.83) | 49.35(41.03-66.35) | 0 |
|  | 140 keV | 74.66(52.81-97.95) | 48.06(39.84-64.41) | 0 |
|  | 150 keV | 73.11(51.67-95.78) | 47.12(38.97-63.08) | 0 |
|  | 160 keV | 71.9(50.83-93.55) | 46.4(38.7-62.07) | 0 |
|  | 170 keV | 71.02(49.97-92.31) | 45.74(37.98-61.28) | 0 |
|  | 180 keV | 70.15(49.42-91.25) | 45.3(37.63-60.58) | 0 |
|  | 190 keV | 69.4(49.04-90.01) | 44.8(37.33-60.09) | 0 |
|  | 200 keV | 68.94(48.7-89.15) | 44.54(37.02-59.66) | 0 |
| **Robust mean absolute deviation** | |  |  |  |
|  | Conventional images | 79.34(45.92-110.11) | 48.9(40.56-67.76) | 0 |
|  | VMIs |  |  |  |
|  | 40 keV | 161.76(83.69-225.06) | 112.66(90.49-162.6) | 0.008 |
|  | 50 keV | 138.44(72.72-198.56) | 82.76(65.85-117.09) | 0 |
|  | 60 keV | 108.32(55.58-149.76) | 64.51(52.65-88.84) | 0 |
|  | 70 keV | 87.88(47.77-119.43) | 51.94(43.5-73.13) | 0 |
|  | 80 keV | 74.11(42.81-102.05) | 45.67(37.51-63.38) | 0 |
|  | 90 keV | 68.04(39.21-91.57) | 40.79(33.78-56) | 0 |
|  | 100 keV | 63.65(37.51-83.48) | 37.39(31.11-51.98) | 0 |
|  | 110 keV | 58.24(34.78-79.09) | 36.14(28.95-49.34) | 0 |
|  | 120 keV | 55.56(33.41-76.12) | 34.94(28.11-46.34) | 0 |
|  | 130 keV | 54.06(32.47-73.67) | 34.17(27.45-45.07) | 0 |
|  | 140 keV | 52.98(31.71-71.63) | 33.37(26.9-43.82) | 0 |
|  | 150 keV | 52.14(31.12-69.92) | 32.73(26.28-42.94) | 0 |
|  | 160 keV | 51(31.83-68.25) | 32.27(26.32-42.5) | 0 |
|  | 170 keV | 49.96(31.42-67.66) | 31.86(25.96-41.85) | 0 |
|  | 180 keV | 49.23(30.13-66.92) | 31.6(25.17-41.35) | 0 |
|  | 190 keV | 48.89(29.75-66.63) | 31.27(25.06-40.96) | 0 |
|  | 200 keV | 48.28(29.38-65.98) | 31.03(24.82-40.66) | 0 |
| **Root mean squared** | |  |  |  |
|  | Conventional images | 1175.24(1080.14-1251.87) | 898.12(670.35-1034.53) | 0 |
|  | VMIs |  |  |  |
|  | 40 keV | 2712.31(2518.15-2896.03) | 2100.15(1574.02-2455.27) | 0 |
|  | 50 keV | 1942.57(1786.4-2092.06) | 1509.08(1116.95-1757.32) | 0 |
|  | 60 keV | 1484.13(1350.77-1585.24) | 1153.37(851.48-1337.68) | 0 |
|  | 70 keV | 1209.55(1098.68-1282.68) | 939.55(691.47-1098.78) | 0 |
|  | 80 keV | 1035.79(938.43-1099.87) | 807.15(591.2-944.39) | 0 |
|  | 90 keV | 918.65(826.34-976.32) | 721.21(525.71-840.94) | 0 |
|  | 100 keV | 837.32(749.52-896.93) | 663.41(481.2-771.63) | 0 |
|  | 110 keV | 780.13(696.2-835.71) | 622.76(449.92-724.74) | 0 |
|  | 120 keV | 739.65(661.33-797.56) | 593.24(427.26-689.49) | 0 |
|  | 130 keV | 710.27(636.1-769.36) | 569.92(410.4-662.84) | 0 |
|  | 140 keV | 685.66(614.17-747.15) | 551.77(397.44-642.35) | 0 |
|  | 150 keV | 666.55(598.03-730.19) | 538.35(387.34-626.35) | 0 |
|  | 160 keV | 652.12(584.61-715.22) | 527.8(379.36-613.71) | 0 |
|  | 170 keV | 641.02(573.53-701.55) | 519.28(372.96-603.55) | 0 |
|  | 180 keV | 631.83(564.28-692.7) | 512.45(367.7-595.19) | 0 |
|  | 190 keV | 623.17(557.21-685.5) | 506.72(363.3-588.25) | 0 |
|  | 200 keV | 615.65(551.4-678.44) | 501.89(359.69-582.57) | 0 |
| **Skewness** | |  |  |  |
|  | Conventional images | -0.73±0.49 | -0.3±0.54 | 0 |
|  | VMIs |  |  |  |
|  | 40 keV | -1.14±0.89 | -0.42±0.8 | 0.000 |
|  | 50 keV | -0.78±0.53 | -0.35±0.66 | 0.000 |
|  | 60 keV | -0.74±0.52 | -0.33±0.62 | 0.000 |
|  | 70 keV | -0.71±0.5 | -0.32±0.57 | 0.000 |
|  | 80 keV | -0.68±0.5 | -0.31±0.55 | 0.000 |
|  | 90 keV | -0.66±0.49 | -0.3±0.52 | 0.000 |
|  | 100 keV | -0.64±0.49 | -0.29±0.51 | 0.000 |
|  | 110 keV | -0.62±0.49 | -0.28±0.5 | 0.000 |
|  | 120 keV | -0.61±0.48 | -0.28±0.49 | 0.000 |
|  | 130 keV | -0.6±0.48 | -0.27±0.49 | 0.000 |
|  | 140 keV | -0.59±0.48 | -0.27±0.49 | 0.000 |
|  | 150 keV | -0.58±0.48 | -0.27±0.49 | 0.000 |
|  | 160 keV | -0.58±0.48 | -0.26±0.49 | 0.000 |
|  | 170 keV | -0.57±0.48 | -0.26±0.48 | 0.000 |
|  | 180 keV | -0.57±0.48 | -0.26±0.48 | 0.000 |
|  | 190 keV | -0.56±0.48 | -0.26±0.48 | 0.000 |
|  | 200 keV | -0.56±0.48 | -0.26±0.48 | 0.000 |
| **Kurtosis** | |  |  |  |
|  | Conventional images | 2.59(2.11-3.34) | 2.81(2.3-3.31) | 0.099 |
|  | VMIs |  |  |  |
|  | 40 keV | 2.86(2.19-4.59) | 2.81(2.27-3.52) | 0.850 |
|  | 50 keV | 2.99±1.32 | 3.09±1.14 | 0.545 |
|  | 60 keV | 2.48(2.08-3.4) | 2.85(2.25-3.37) | 0.067 |
|  | 70 keV | 2.5(2.07-3.32) | 2.82(2.29-3.39) | 0.037 |
|  | 80 keV | 2.5(2.03-3.27) | 2.79(2.32-3.32) | 0.020 |
|  | 90 keV | 2.48(2.02-3.23) | 2.79(2.34-3.38) | 0.011 |
|  | 100 keV | 2.46(2-3.19) | 2.78(2.34-3.38) | 0.007 |
|  | 110 keV | 2.44(1.99-3.12) | 2.75(2.36-3.32) | 0.004 |
|  | 120 keV | 2.43(2-3.15) | 2.74(2.36-3.3) | 0.002 |
|  | 130 keV | 2.42(1.99-3.08) | 2.74(2.34-3.32) | 0.001 |
|  | 140 keV | 2.43(2-3.09) | 2.74(2.35-3.31) | 0 |
|  | 150 keV | 2.79±1.21 | 3±0.85 | 0.164 |
|  | 160 keV | 2.79±1.21 | 3±0.85 | 0.148 |
|  | 170 keV | 2.78±1.21 | 3±0.85 | 0.135 |
|  | 180 keV | 2.78±1.2 | 3.01±0.85 | 0.125 |
|  | 190 keV | 2.78±1.2 | 3.01±0.85 | 0.120 |
|  | 200 keV | 2.77±1.2 | 3.01±0.85 | 0.110 |
| **Variance** | |  |  |  |
|  | Conventional images | 18663.47(8173.84-32268.86) | 8261.05(5132.98-13524.75) | 0 |
|  | VMIs |  |  |  |
|  | 40 keV | 78747.39(32750.83-164753.06) | 46454.58(23881.2-73876.61) | 0 |
|  | 50 keV | 52860.38(20154.44-94041.91) | 23749.03(13717.79-38218.41) | 0 |
|  | 60 keV | 31347.09(12718.75-55353.24) | 14321.65(8575.01-22578.83) | 0 |
|  | 70 keV | 22116.9(9329.8-36328.28) | 9817.82(5919.99-15231.74) | 0 |
|  | 80 keV | 16592.22(7605.35-27570.39) | 7261.32(4657.42-11498.85) | 0 |
|  | 90 keV | 13344.41(6455.2-21917.39) | 5893.96(3852.45-9694.33) | 0 |
|  | 100 keV | 11344.08(5820.8-18742.76) | 5242.89(3267.58-8451.12) | 0 |
|  | 110 keV | 10210.72(5324.35-16676.69) | 4575.1(2962.63-7457.32) | 0 |
|  | 120 keV | 9216.9(4988.91-15363.89) | 4260.26(2846.75-6902.47) | 0 |
|  | 130 keV | 8619.31(4666.92-14235.8) | 4040.71(2616.54-6531.84) | 0 |
|  | 140 keV | 8282.86(4440.41-13319.47) | 3901.01(2519.66-6258.78) | 0 |
|  | 150 keV | 7817.81(4239.35-12683.57) | 3786.22(2457.95-6055.35) | 0 |
|  | 160 keV | 7443.95(4065.49-12112.53) | 3668.05(2420.59-5885.53) | 0 |
|  | 170 keV | 7163.04(3939.07-11687.76) | 3566.63(2346.14-5750.44) | 0 |
|  | 180 keV | 6961.89(3851.8-11421.69) | 3500.3(2278.14-5675.5) | 0 |
|  | 190 keV | 6813.31(3755.67-11295.75) | 3458.34(2243.92-5605.08) | 0 |
|  | 200 keV | 78747.39(32750.83-164753.06) | 46454.58(23881.2-73876.61) | 0 |
| **Uniformity** | |  |  |  |
|  | Conventional images | 0.05(0.05-0.06) | 0.03(0.02-0.04) | 0 |
|  | VMIs |  |  | 0 |
|  | 40 keV | 0.08(0.05-0.24) | 0.02(0.02-0.03) | 0 |
|  | 50 keV | 0.05(0.05-0.06) | 0.03(0.02-0.04) | 0 |
|  | 60 keV | 0.05(0.05-0.06) | 0.03(0.02-0.04) | 0 |
|  | 70 keV | 0.06(0.05-0.06) | 0.03(0.02-0.04) | 0 |
|  | 80 keV | 0.06±0.02 | 0.03±0.01 | 0 |
|  | 90 keV | 0.06(0.05-0.07) | 0.04(0.03-0.04) | 0 |
|  | 100 keV | 0.06(0.05-0.07) | 0.04(0.03-0.04) | 0 |
|  | 110 keV | 0.06±0.02 | 0.04±0.02 | 0.000 |
|  | 120 keV | 0.06±0.02 | 0.04±0.01 | 0.000 |
|  | 130 keV | 0.06±0.02 | 0.04±0.01 | 0.000 |
|  | 140 keV | 0.06±0.02 | 0.04±0.02 | 0.000 |
|  | 150 keV | 0.06±0.02 | 0.04±0.01 | 0.000 |
|  | 160 keV | 0.06±0.02 | 0.04±0.02 | 0.000 |
|  | 170 keV | 0.06±0.02 | 0.04±0.02 | 0.000 |
|  | 180 keV | 0.06±0.02 | 0.04±0.02 | 0.000 |
|  | 190 keV | 0.06±0.02 | 0.04±0.02 | 0.000 |
|  | 200 keV | 0.06±0.02 | 0.04±0.02 | 0.000 |

Supplementary table 3. Diagnostic performance of first-order feature parameters of spectral images in differentiating between BI and OBM

| **Parameters** | | Cutoff | AUC (95%CI) | Sensitivity (95%CI) | Specificity (95%CI) |
| --- | --- | --- | --- | --- | --- |
| **Entropy** | |  |  |  |  |
|  | Conventional images | 4.632 | 0.91 [0.87, 0.94] | 87 (78/91) [81, 94] | 82 (103/125) [75,89] |
|  | VMIs |  |  |  |  |
|  | 40 keV | 4.593 | 0.93 [0.89, 0.97] | 92 (84/91) [86, 97] | 88 (110/125) [82, 93] |
|  | 50 keV | 4.590 | 0.91 [0.88, 0.95] | 93 (85/91) [88, 98] | 77 (97/125) [70, 84] |
|  | 60 keV | 4.638 | 0.91 [0.88, 0.95] | 90 (82/91) [84, 96] | 80 (101/125) [73, 87] |
|  | 70 keV | 4.586 | 0.90 [0.86, 0.94] | 89 (81/91) [82, 95] | 80 (100/125) [73, 87] |
|  | 80 keV | 4.493 | 0.91 [0.87, 0.94] | 91 (83/91) [85, 97] | 80 (100/125) [73, 87] |
|  | 90 keV | 4.547 | 0.89 [0.84, 0.93] | 83 (76/91) [75, 91] | 83 (104/125) [76, 89] |
|  | 100 keV | 4.547 | 0.89 [0.85, 0.93] | 83 (76/91) [75, 91] | 84 (105/125) [77, 90] |
|  | 110 keV | 4.425 | 0.88 [0.83, 0.93] | 86 (79/91) [79, 93] | 80 (101/125) [73, 87] |
|  | 120 keV | 4.422 | 0.88 [0.84, 0.93] | 86 (79/91) [79, 93] | 78 (98/125) [70, 85] |
|  | 130 keV | 4.555 | 0.86 [0.81, 0.91] | 76 (70/91) [68, 85] | 84 (106/125) [78, 90] |
|  | 140 keV | 4.439 | 0.87 [0.82, 0.92] | 82 (75/91) [74, 90] | 82 (103/125) [75, 88] |
|  | 150 keV | 4.42 | 0.87 [0.82, 0.92] | 82 (75/91) [74, 90] | 81 (102/125) [74, 88] |
|  | 160 keV | 4.434 | 0.87 [0.82, 0.91] | 82 (75/91) [74, 90] | 81 (102/125) [74, 88] |
|  | 170 keV | 4.51 | 0.87 [0.82, 0.92] | 76 (70/91) [68, 85] | 86 (108/125) [80, 92] |
|  | 180 keV | 4.426 | 0.87 [0.82, 0.92] | 82 (75/91) [74, 90] | 82 (103/125) [75, 88] |
|  | 190 keV | 4.42 | 0.88 [0.83, 0.92] | 81 (74/91) [73, 89] | 83 (105/125) [77, 90] |
|  | 200 keV | 4.449 | 0.87 [0.82, 0.92] | 79 (72/91) [70, 87] | 84 (106/125) [78, 90] |
| **Minimum** | |  |  |  |  |
|  | Conventional images | 650 | 0.73 [0.66, 0.80] | 81 (74/91) [74, 88] | 56 (70/125) [45, 66] |
|  | VMIs |  |  |  |  |
|  | 40 keV | 1460.5 | 0.71 [0.64, 0.78] | 84 (76/91) [77, 90] | 52 (66/125) [42, 63] |
|  | 50 keV | 1030 | 0.71 [0.64, 0.78] | 83 (76/91) [76, 89] | 52 (66/125) [42, 63] |
|  | 60 keV | 816 | 0.71 [0.64, 0.78] | 81 (74/91) [74, 88] | 53 (67/125) [43, 64] |
|  | 70 keV | 662.5 | 0.71 [0.64, 0.78] | 81 (74/91) [74, 88] | 54 (69/125) [44, 65] |
|  | 80 keV | 570.5 | 0.71 [0.64, 0.78] | 80 (74/91) [73, 87] | 53 (67/125) [43, 64] |
|  | 90 keV | 504.5 | 0.71 [0.64, 0.78] | 80 (74/91) [73, 87] | 53 (67/125) [43, 64] |
|  | 100 keV | 451.5 | 0.71 [0.64, 0.78] | 81 (74/91) [74, 88] | 52 (66/125) [42, 63] |
|  | 110 keV | 417.5 | 0.71 [0.64, 0.77] | 81 (74/91) [74, 88] | 52 (66/125) [42, 63] |
|  | 120 keV | 393 | 0.71 [0.64, 0.78] | 81 (74/91) [74, 88] | 52 (66/125) [42, 63] |
|  | 130 keV | 374.5 | 0.71 [0.64, 0.78] | 80 (73/91) [73, 87] | 52 (66/125) [42, 63] |
|  | 140 keV | 355.5 | 0.71 [0.64, 0.78] | 81 (74/91) [74, 88] | 51 (65/125) [41, 61] |
|  | 150 keV | 352 | 0.71 [0.64, 0.78] | 79 (73/91) [72, 86] | 52 (66/125) [42, 63] |
|  | 160 keV | 343.5 | 0.71 [0.64, 0.78] | 79 (73/91) [72, 86] | 52 (66/125) [42, 63] |
|  | 170 keV | 316.5 | 0.71 [0.64, 0.78] | 83 (76/91) [77, 90] | 48 (63/125) [38, 58] |
|  | 180 keV | 311.5 | 0.71 [0.64, 0.78] | 83 (76/91) [77, 90] | 48 (63/125) [38, 58] |
|  | 190 keV | 307 | 0.71 [0.64, 0.77] | 83 (76/91) [77, 90] | 48 (63/125) [38, 58] |
|  | 200 keV | 303.5 | 0.71 [0.64, 0.78] | 83 (76/91) [77, 90] | 48 (63/125) [38, 58] |
| **Maximum** | |  |  |  |  |
|  | Conventional images | 1301 | 0.86 [0.81, 0.91] | 72 (66/91) [64, 79] | 90 (113/125) [84, 96] |
|  | VMIs |  |  |  |  |
|  | 40 keV | 2893 | 0.83 [0.78, 0.89] | 79 (72/91) [72, 86] | 81 (102/125) [73, 89] |
|  | 50 keV | 2074 | 0.84 [0.78, 0.89] | 80 (72.8/91) [73, 87] | 79 (99/125) [70, 87] |
|  | 60 keV | 1604 | 0.84 [0.78, 0.89] | 76 (69/91) [68, 83] | 81 (102/125) [73, 89] |
|  | 70 keV | 1310 | 0.83 [0.78, 0.89] | 76 (69/91) [68, 83] | 79 (99/125) [70, 87] |
|  | 80 keV | 1135.5 | 0.83 [0.78, 0.88] | 74 (68/91) [66, 82] | 81 (102/125) [73, 89] |
|  | 90 keV | 1008.5 | 0.82 [0.77, 0.88] | 72 (66/91) [65, 80] | 80 (100/125) [72, 88] |
|  | 100 keV | 917.5 | 0.81 [0.76, 0.87] | 75 (68/91) [67, 82] | 76 (96/125) [68, 85] |
|  | 110 keV | 857 | 0.81 [0.75, 0.86] | 74 (67/91) [66, 81] | 75 (95/125) [67, 84] |
|  | 120 keV | 814.5 | 0.80 [0.74, 0.86] | 74 (67/91) [66, 81] | 74 (93/125) [65, 83] |
|  | 130 keV | 779.5 | 0.80 [0.74, 0.86] | 74 (67/91) [66, 81] | 73 (92/125) [64, 82] |
|  | 140 keV | 747.5 | 0.8 [0.74, 0.85] | 76 (70/91) [68, 83] | 71 (89/125) [62, 80] |
|  | 150 keV | 731.5 | 0.79 [0.73, 0.85] | 73 (67/91) [65, 81] | 72 (91/125) [63, 81] |
|  | 160 keV | 714.5 | 0.79 [0.73, 0.85] | 75 (69/91) [68, 83] | 69 (87/125) [59, 78] |
|  | 170 keV | 666.5 | 0.79 [0.73, 0.85] | 88 (81/91) [83, 94] | 56 (70/125) [45, 66] |
|  | 180 keV | 657.5 | 0.79 [0.73, 0.85] | 88 (81/91) [83, 94] | 56 (70/125) [45, 66] |
|  | 190 keV | 650.5 | 0.78 [0.72, 0.84] | 87 (80/91) [82, 93] | 56 (70/125) [45, 66] |
|  | 200 keV | 709.5 | 0.78 [0.72, 0.84] | 61 (56/91) [53, 70] | 81 (102/125) [73, 89] |
| **10^th^ percentile** | |  |  |  |  |
|  | Conventional images | 939.45 | 0.77 [0.70, 0.83] | 60 (55/91) [52, 69] | 82 (103/125) [74, 90] |
|  | VMIs |  |  |  |  |
|  | 40 keV | 1847.1 | 0.74 [0.67, 0.81] | 84 (76/91) [77, 90] | 56 (70/125) [45, 66] |
|  | 50 keV | 1292.85 | 0.74 [0.67, 0.81] | 84 (77/91) [78, 91] | 53 (67/125) [43, 64] |
|  | 60 keV | 1015.5 | 0.74 [0.67, 0.80] | 79 (72/91) [72, 86] | 57 (71/125) [47, 67] |
|  | 70 keV | 815.7 | 0.73 [0.67, 0.80] | 80 (74/91) [73, 87] | 54 (69/125) [44, 65] |
|  | 80 keV | 574.7 | 0.73 [0.66, 0.80] | 90 (82/91) [85, 95] | 45 (56/125) [34, 55] |
|  | 90 keV | 509.8 | 0.73 [0.66, 0.79] | 90 (82/91) [85, 95] | 45 (56/125) [34, 55] |
|  | 100 keV | 464.45 | 0.72 [0.66, 0.79] | 90 (82/91) [85, 95] | 45 (56/125) [34, 55] |
|  | 110 keV | 433.9 | 0.72 [0.65, 0.79] | 89 (81/91) [84, 94] | 45 (56/125) [34, 55] |
|  | 120 keV | 406.5 | 0.72 [0.65, 0.79] | 90 (82/91) [85, 95] | 44 (55/125) [33, 54] |
|  | 130 keV | 388.15 | 0.72 [0.65, 0.79] | 90 (82/91) [85, 95] | 44 (55/125) [33, 54] |
|  | 140 keV | 358.8 | 0.72 [0.65, 0.78] | 92 (84/91) [88, 97] | 41 (52/125) [31, 51] |
|  | 150 keV | 350 | 0.71 [0.65, 0.78] | 92 (84/91) [88, 97] | 41 (52/125) [31, 51] |
|  | 160 keV | 343 | 0.71 [0.65, 0.78] | 92 (84/91) [88, 97] | 41 (52/125) [31, 51] |
|  | 170 keV | 337.5 | 0.71 [0.64, 0.78] | 92 (84/91) [88, 97] | 41 (52/125) [31, 51] |
|  | 180 keV | 333 | 0.71 [0.64, 0.78] | 92 (84/91) [88, 97] | 41 (52/125) [31, 51] |
|  | 190 keV | 329 | 0.71 [0.64, 0.78] | 92 (84/91) [88, 97] | 41 (52/125) [31, 51] |
|  | 200 keV | 325.7 | 0.71 [0.64, 0.78] | 92 (84/91) [88, 97] | 41 (52/125) [31, 51] |
| **90^th^ percentile** | |  |  |  |  |
|  | Conventional images | 1161.85 | 0.89 [0.84, 0.93] | 87 (79/91) [81, 93] | 81 (102/125) [73, 89] |
|  | VMIs |  |  |  |  |
|  | 40 keV | 2766.95 | 0.87 [0.82, 0.92] | 84 (76/91) [77, 90] | 82 (103/125) [74, 90] |
|  | 50 keV | 1980.75 | 0.87 [0.81, 0.92] | 82 (75/91) [75, 89] | 84 (106/125) [77, 92] |
|  | 60 keV | 1520.95 | 0.86 [0.81, 0.91] | 81 (74/91) [74, 88] | 83 (104/125) [75, 91] |
|  | 70 keV | 1219.7 | 0.86 [0.81, 0.91] | 83 (76/91) [76, 89] | 81 (102/125) [73, 89] |
|  | 80 keV | 1050.3 | 0.86 [0.81, 0.91] | 80 (74/91) [73, 87] | 81 (102/125) [73, 89] |
|  | 90 keV | 948.75 | 0.86 [0.81, 0.91] | 78 (71/91) [71, 85] | 83 (104/125) [75, 91] |
|  | 100 keV | 871.35 | 0.85 [0.80, 0.90] | 76 (70/91) [69, 84] | 83 (104/125) [75, 91] |
|  | 110 keV | 811.55 | 0.85 [0.80, 0.90] | 78 (71/91) [70, 85] | 81 (102/125) [73, 89] |
|  | 120 keV | 790.45 | 0.85 [0.80, 0.90] | 73 (67/91) [65, 81] | 85 (107/125) [78, 92] |
|  | 130 keV | 756.1 | 0.84 [0.79, 0.9] | 73 (67/91) [65, 81] | 83 (104/125) [75, 91] |
|  | 140 keV | 724.4 | 0.84 [0.79, 0.89] | 75 (69/91) [68, 83] | 80 (100/125) [72, 88] |
|  | 150 keV | 706.45 | 0.84 [0.79, 0.89] | 75 (69/91) [68, 83] | 79 (99/125) [70, 87] |
|  | 160 keV | 688.25 | 0.84 [0.79, 0.89] | 75 (69/91) [68, 83] | 78 (98/125) [69, 86] |
|  | 170 keV | 685.7 | 0.84 [0.79, 0.89] | 70 (64/91) [62, 78] | 82 (103/125) [74, 90] |
|  | 180 keV | 668.1 | 0.84 [0.78, 0.89] | 74 (68/91) [67, 82] | 79 (99/125) [70, 87] |
|  | 190 keV | 660.8 | 0.84 [0.78, 0.89] | 74 (67/91) [66, 81] | 79 (99/125) [70, 87] |
|  | 200 keV | 646.4 | 0.84 [0.78, 0.89] | 77 (70/91) [69, 84] | 75 (95/125) [67, 84] |
| **Mean** | |  |  |  |  |
|  | Conventional images | 1050.704 | 0.85 [0.80, 0.90] | 78 (71/91) [71, 85] | 83 (104/125) [75, 91] |
|  | VMIs |  |  |  |  |
|  | 40 keV | 2454.291 | 0.83 [0.77, 0.89] | 78 (71/91) [71, 85] | 78 (98/125) [69, 86] |
|  | 50 keV | 1795.979 | 0.83 [0.77, 0.89] | 72 (66/91) [65, 80] | 83 (104/125) [75, 91] |
|  | 60 keV | 1364.857 | 0.83 [0.77, 0.88] | 73 (67/91) [65, 81] | 81 (102/125) [73, 89] |
|  | 70 keV | 1147.503 | 0.82 [0.76, 0.88] | 64 (58/91) [55, 72] | 89 (111/125) [82, 95] |
|  | 80 keV | 974.971 | 0.82 [0.76, 0.87] | 64 (58/91) [55, 72] | 87 (110/125) [81, 94] |
|  | 90 keV | 854.919 | 0.81 [0.76, 0.87] | 66 (60/91) [58, 74] | 83 (104/125) [75, 91] |
|  | 100 keV | 785.498 | 0.81 [0.75, 0.87] | 64 (59/91) [56, 73] | 84 (106/125) [77, 92] |
|  | 110 keV | 734.392 | 0.81 [0.75, 0.86] | 63 (58/91) [54, 71] | 83 (104/125) [75, 91] |
|  | 120 keV | 650.046 | 0.80 [0.75, 0.86] | 78 (71/91) [70, 85] | 68 (85/125) [58, 77] |
|  | 130 keV | 623.191 | 0.80 [0.74, 0.86] | 78 (71/91) [70, 85] | 68 (85/125) [58, 77] |
|  | 140 keV | 602.03 | 0.80 [0.74, 0.86] | 78 (71/91) [70, 85] | 68 (85/125) [58, 77] |
|  | 150 keV | 585.593 | 0.80 [0.74, 0.85] | 77 (70/91) [69, 84] | 68 (85/125) [58, 77] |
|  | 160 keV | 577.081 | 0.79 [0.74, 0.85] | 75 (69/91) [68, 83] | 69 (87/125) [59, 78] |
|  | 170 keV | 564.717 | 0.79 [0.73, 0.85] | 76 (70/91) [68, 83] | 69 (87/125) [59, 78] |
|  | 180 keV | 555.968 | 0.79 [0.73, 0.85] | 76 (70/91) [68, 83] | 69 (87/125) [59, 78] |
|  | 190 keV | 549.222 | 0.79 [0.73, 0.85] | 76 (70/91) [68, 83] | 69 (87/125) [59, 78] |
|  | 200 keV | 543.783 | 0.79 [0.73, 0.85] | 76 (70/91) [68, 83] | 69 (87/125) [59, 78] |
| **Median** | |  |  |  |  |
|  | Conventional images | 1067 | 0.87 [0.82, 0.92] | 82 (75/91) [75, 89] | 83 (104/125) [75, 91] |
|  | VMIs |  |  |  |  |
|  | 40 keV | 2516.75 | 0.85 [0.80, 0.90] | 78 (71/91) [71, 85] | 83 (104/125) [75, 91] |
|  | 50 keV | 1811.5 | 0.85 [0.79, 0.90] | 76 (70/91) [69, 84] | 83 (104/125) [75, 91] |
|  | 60 keV | 1409.5 | 0.84 [0.79, 0.90] | 72 (66/91) [65, 80] | 86 (109/125) [79, 93] |
|  | 70 keV | 1147.25 | 0.84 [0.79, 0.89] | 69 (63/91) [61, 77] | 87 (110/125) [81, 94] |
|  | 80 keV | 956.5 | 0.84 [0.78, 0.89] | 74 (68/91) [66, 82] | 81 (102/125) [73, 89] |
|  | 90 keV | 869.75 | 0.83 [0.78, 0.88] | 68 (62/91) [59, 76] | 84 (106/125) [77, 92] |
|  | 100 keV | 788.75 | 0.83 [0.77, 0.88] | 68 (63/91) [60, 76] | 83 (104/125) [75, 91] |
|  | 110 keV | 680.25 | 0.82 [0.76, 0.87] | 83 (76/91) [77, 90] | 65 (82/125) [56, 75] |
|  | 120 keV | 707.5 | 0.82 [0.76, 0.87] | 64 (58/91) [55, 72] | 84 (106/125) [77, 92] |
|  | 130 keV | 621.25 | 0.81 [0.76, 0.87] | 82 (75/91) [75, 88] | 65 (82/125) [56, 75] |
|  | 140 keV | 598.5 | 0.81 [0.75, 0.87] | 82 (75/91) [75, 88] | 65 (82/125) [56, 75] |
|  | 150 keV | 581.5 | 0.81 [0.75, 0.87] | 82 (75/91) [75, 88] | 65 (82/125) [56, 75] |
|  | 160 keV | 573.5 | 0.81 [0.75, 0.86] | 81 (74/91) [74, 88] | 65 (82/125) [56, 75] |
|  | 170 keV | 562.75 | 0.81 [0.75, 0.86] | 81 (74/91) [74, 88] | 65 (82/125) [56, 75] |
|  | 180 keV | 554.5 | 0.81 [0.75, 0.86] | 81 (74/91) [74, 88] | 65 (82/125) [56, 75] |
|  | 190 keV | 547.5 | 0.81 [0.75, 0.86] | 81 (74/91) [74, 88] | 65 (82/125) [56, 75] |
|  | 200 keV | 549 | 0.80 [0.75, 0.86] | 78 (72/91) [71, 86] | 67 (84/125) [57, 76] |
| **Interquartile range** | |  |  |  |  |
|  | Conventional images | 140.625 | 0.68 [0.60, 0.75] | 66 (60/91) [58, 74] | 69 (87/125) [59, 78] |
|  | VMIs |  |  |  |  |
|  | 40 keV | 494.375 | 0.59 [0.51, 0.66] | 33 (31/91) [25, 41] | 92 (115/125) [86, 97] |
|  | 50 keV | 362 | 0.67 [0.60, 0.74] | 38 (35/91) [29, 46] | 93 (117/125) [88, 98] |
|  | 60 keV | 181.125 | 0.68 [0.61, 0.75] | 67 (61/91) [59, 75] | 69 (87/125) [59, 78] |
|  | 70 keV | 157.375 | 0.69 [0.62, 0.76] | 67 (61/91) [59, 75] | 71 (89/125) [62, 80] |
|  | 80 keV | 137.5 | 0.69 [0.63, 0.76] | 68 (62/91) [59, 76] | 72 (91/125) [63, 81] |
|  | 90 keV | 124.5 | 0.70 [0.63, 0.77] | 68 (63/91) [60, 76] | 70 (88/125) [60, 79] |
|  | 100 keV | 122.75 | 0.70 [0.63, 0.77] | 64 (59/91) [56, 73] | 74 (93/125) [65, 83] |
|  | 110 keV | 116.75 | 0.70 [0.63, 0.77] | 64 (58/91) [55, 72] | 75 (95/125) [67, 84] |
|  | 120 keV | 113.5 | 0.70 [0.63, 0.77] | 63 (58/91) [54, 71] | 75 (95/125) [67, 84] |
|  | 130 keV | 104.5 | 0.70 [0.63, 0.77] | 65 (60/91) [57, 74] | 72 (91/125) [63, 81] |
|  | 140 keV | 108.5 | 0.70 [0.63, 0.77] | 63 (58/91) [54, 71] | 75 (95/125) [67, 84] |
|  | 150 keV | 106 | 0.70 [0.63, 0.77] | 63 (58/91) [54, 71] | 75 (95/125) [67, 84] |
|  | 160 keV | 103.5 | 0.70 [0.63, 0.77] | 64 (58/91) [55, 72] | 75 (95/125) [67, 84] |
|  | 170 keV | 102.5 | 0.70 [0.63, 0.77] | 63 (58/91) [54, 71] | 75 (95/125) [67, 84] |
|  | 180 keV | 102.5 | 0.70 [0.63, 0.77] | 63 (58/91) [54, 71] | 75 (95/125) [67, 84] |
|  | 190 keV | 101.875 | 0.7 [0.63, 0.77] | 63 (58/91) [54, 71] | 75 (95/125) [67, 84] |
|  | 200 keV | 101.375 | 0.70 [0.63, 0.77] | 63 (58/91) [54, 71] | 75 (95/125) [67, 84] |
| **Range** | |  |  |  |  |
|  | Conventional images | 513.5 | 0.59 [0.52, 0.67] | 48 (44/91) [39, 56] | 74 (93/125) [65, 83] |
|  | VMIs |  |  |  |  |
|  | 50 keV | 751 | 0.59 [0.52, 0.67] | 56 (52/91) [48, 65] | 65 (82/125) [56, 75] |
|  | 60 keV | 605.5 | 0.60 [0.52, 0.67] | 55 (50/91) [46, 63] | 69 (87/125) [59, 78] |
|  | 70 keV | 513.5 | 0.60 [0.52, 0.67] | 53 (49/91) [44, 62] | 70 (88/125) [60, 79] |
|  | 80 keV | 435.5 | 0.59 [0.52, 0.67] | 57 (52/91) [48, 66] | 68 (85/125) [58, 77] |
|  | 90 keV | 390.5 | 0.59 [0.51, 0.67] | 57 (52/91) [48, 66] | 67 (84/125) [57, 76] |
|  | 100 keV | 366.5 | 0.59 [0.51, 0.66] | 56 (52/91) [48, 65] | 67 (84/125) [57, 76] |
|  | 110 keV | 364 | 0.58 [0.50, 0.66] | 49 (45/91) [40, 58] | 71 (90/125) [62, 80] |
|  | 120 keV | 346.5 | 0.58 [0.50, 0.65] | 49 (45/91) [40, 58] | 71 (90/125) [62, 80] |
| **Mean absolute deviation** | |  |  |  |  |
|  | Conventional images | 122.637 | 0.70 [0.63, 0.77] | 46 (42/91) [37, 55] | 92 (115/125) [86, 97] |
|  | VMIs |  |  |  |  |
|  | 40 keV | 202.374 | 0.63 [0.56, 0.71] | 61 (56/91) [53, 70] | 69 (87/125) [59, 78] |
|  | 50 keV | 178.502 | 0.70 [0.63, 0.77] | 54 (50/91) [45, 63] | 83 (104/125) [75, 91] |
|  | 60 keV | 122.445 | 0.70 [0.63, 0.77] | 63 (58/91) [54, 71] | 75 (95/125) [67, 84] |
|  | 70 keV | 111.47 | 0.71 [0.64, 0.78] | 56 (52/91) [48, 65] | 82 (103/125) [74, 90] |
|  | 80 keV | 95.366 | 0.71 [0.64, 0.78] | 58 (53/91) [49, 67] | 81 (102/125) [73, 89] |
|  | 90 keV | 86.618 | 0.71 [0.64, 0.78] | 57 (52/91) [48, 66] | 82 (103/125) [74, 90] |
|  | 100 keV | 69.69 | 0.71 [0.64, 0.78] | 67 (61/91) [59, 75] | 72 (91/125) [63, 81] |
|  | 110 keV | 75.895 | 0.71 [0.64, 0.78] | 56 (52/91) [48, 65] | 82 (103/125) [74, 90] |
|  | 120 keV | 59.676 | 0.71 [0.64, 0.78] | 73 (67/91) [65, 81] | 67 (84/125) [57, 76] |
|  | 130 keV | 59.938 | 0.71 [0.64, 0.78] | 70 (64/91) [62, 78] | 69 (87/125) [59, 78] |
|  | 140 keV | 59.026 | 0.71 [0.64, 0.78] | 70 (64/91) [62, 78] | 70 (88/125) [60, 79] |
|  | 150 keV | 58.083 | 0.71 [0.64, 0.78] | 70 (64/91) [62, 78] | 70 (88/125) [60, 79] |
|  | 160 keV | 57.384 | 0.71 [0.64, 0.78] | 70 (64/91) [62, 78] | 70 (88/125) [60, 79] |
|  | 170 keV | 56.729 | 0.71 [0.64, 0.78] | 70 (64/91) [62, 78] | 70 (88/125) [60, 79] |
|  | 180 keV | 56.302 | 0.71 [0.64, 0.78] | 69 (64/91) [61, 78] | 70 (88/125) [60, 79] |
|  | 190 keV | 56.016 | 0.71 [0.64, 0.78] | 69 (64/91) [61, 78] | 70 (88/125) [60, 79] |
|  | 200 keV | 55.798 | 0.71 [0.64, 0.78] | 69 (64/91) [61, 78] | 70 (88/125) [60, 79] |
| **Robust mean absolute deviation** | |  |  |  |  |
|  | Conventional images | 63.589 | 0.68 [0.61, 0.75] | 63 (58/91) [54, 71] | 73 (92/125) [64, 82] |
|  | VMIs |  |  |  |  |
|  | 40 keV | 193.222 | 0.60 [0.53, 0.68] | 41 (38/91) [33, 50] | 86 (109/125) [79, 93] |
|  | 50 keV | 136.615 | 0.68 [0.61, 0.75] | 51 (47/91) [42, 60] | 86 (109/125) [79, 93] |
|  | 60 keV | 111.638 | 0.68 [0.61, 0.75] | 48 (44/91) [40, 57] | 89 (111/125) [82, 95] |
|  | 70 keV | 93.378 | 0.69 [0.62, 0.76] | 48 (44/91) [39, 56] | 90 (113/125) [84, 96] |
|  | 80 keV | 80.173 | 0.69 [0.62, 0.76] | 49 (45/91) [40, 58] | 90 (113/125) [84, 96] |
|  | 90 keV | 52.553 | 0.7 [0.62, 0.77] | 67 (61/91) [59, 75] | 72 (91/125) [63, 81] |
|  | 100 keV | 49.963 | 0.70 [0.63, 0.77] | 66 (60/91) [58, 74] | 73 (92/125) [64, 82] |
|  | 110 keV | 46.974 | 0.70 [0.63, 0.77] | 65 (60/91) [57, 74] | 73 (92/125) [64, 82] |
|  | 120 keV | 47.362 | 0.7 [0.63, 0.77] | 64 (58/91) [55, 72] | 76 (96/125) [68, 85] |
|  | 130 keV | 45.828 | 0.70 [0.63, 0.77] | 64 (58/91) [55, 72] | 78 (98/125) [69, 86] |
|  | 140 keV | 44.921 | 0.70 [0.63, 0.77] | 64 (58/91) [55, 72] | 78 (98/125) [69, 86] |
|  | 150 keV | 43.336 | 0.70 [0.63, 0.77] | 65 (60/91) [57, 74] | 76 (96/125) [68, 85] |
|  | 160 keV | 42.84 | 0.70 [0.63, 0.77] | 64 (58/91) [55, 72] | 76 (96/125) [68, 85] |
|  | 170 keV | 41.878 | 0.70 [0.63, 0.77] | 65 (59/91) [56, 73] | 76 (96/125) [68, 85] |
|  | 180 keV | 41.446 | 0.70 [0.63, 0.77] | 64 (58/91) [55, 72] | 76 (96/125) [68, 85] |
|  | 190 keV | 41.672 | 0.70 [0.63, 0.77] | 62 (57/91) [54, 71] | 78 (98/125) [69, 86] |
|  | 200 keV | 41.348 | 0.70 [0.63, 0.77] | 63 (58/91) [54, 71] | 76 (96/125) [68, 85] |
| **Root mean squared** | |  |  |  |  |
|  | Conventional images | 1057.994 | 0.86 [0.81, 0.91] | 79 (72/91) [72, 86] | 83 (104/125) [75, 91] |
|  | VMIs |  |  |  |  |
|  | 40 keV | 2475.697 | 0.84 [0.78, 0.89] | 79 (72/91) [72, 86] | 79 (99/125) [70, 87] |
|  | 50 keV | 1781.82 | 0.84 [0.78, 0.89] | 76 (69/91) [68, 83] | 81 (102/125) [73, 89] |
|  | 60 keV | 1377.794 | 0.83 [0.78, 0.89] | 73 (67/91) [65, 81] | 83 (104/125) [75, 91] |
|  | 70 keV | 1127.755 | 0.83 [0.77, 0.88] | 72 (66/91) [64, 79] | 83 (104/125) [75, 91] |
|  | 80 keV | 980.953 | 0.83 [0.77, 0.88] | 64 (59/91) [56, 73] | 89 (111/125) [82, 95] |
|  | 90 keV | 856.732 | 0.82 [0.77, 0.88] | 68 (63/91) [60, 76] | 82 (103/125) [74, 90] |
|  | 100 keV | 789.061 | 0.82 [0.76, 0.87] | 66 (60/91) [58, 74] | 84 (106/125) [77, 92] |
|  | 110 keV | 688.855 | 0.81 [0.76, 0.87] | 80 (73/91) [73, 87] | 68 (85/125) [58, 77] |
|  | 120 keV | 707.602 | 0.81 [0.75, 0.87] | 61 (56/91) [53, 70] | 85 (107/125) [78, 92] |
|  | 130 keV | 626.122 | 0.81 [0.75, 0.86] | 78 (72/91) [71, 86] | 68 (85/125) [58, 77] |
|  | 140 keV | 605.651 | 0.81 [0.75, 0.86] | 78 (72/91) [71, 86] | 68 (85/125) [58, 77] |
|  | 150 keV | 593.907 | 0.80 [0.75, 0.86] | 77 (70/91) [69, 84] | 69 (87/125) [59, 78] |
|  | 160 keV | 580.758 | 0.80 [0.74, 0.86] | 77 (70/91) [69, 84] | 69 (87/125) [59, 78] |
|  | 170 keV | 570.478 | 0.80 [0.74, 0.86] | 77 (70/91) [69, 84] | 69 (87/125) [59, 78] |
|  | 180 keV | 619.003 | 0.80 [0.74, 0.86] | 56 (52/91) [48, 65] | 89 (111/125) [82, 95] |
|  | 190 keV | 556.533 | 0.80 [0.74, 0.86] | 76 (70/91) [68, 83] | 69 (87/125) [59, 78] |
|  | 200 keV | 606.471 | 0.80 [0.74, 0.86] | 56 (51/91) [47, 64] | 89 (111/125) [82, 95] |
| **Skewness** | |  |  |  |  |
|  | Conventional images | -0.236 | 0.73 [0.66, 0.80] | 54 (50/91) [44, 65] | 87 (109/125) [81, 93] |
|  | VMIs |  |  |  |  |
|  | 40 keV | -0.495 | 0.74 [0.67, 0.81] | 62 (57/91) [52, 72] | 80 (100/125) [73, 87] |
|  | 50 keV | -0.441 | 0.70 [0.63, 0.77] | 60 (55/91) [50, 70] | 76 (95/125) [68, 83] |
|  | 60 keV | -0.268 | 0.70 [0.63, 0.77] | 53 (49/91) [43, 64] | 84 (106/125) [78, 91] |
|  | 70 keV | -0.274 | 0.70 [0.63, 0.77] | 57 (52/91) [47, 67] | 83 (104/125) [76, 89] |
|  | 80 keV | -0.237 | 0.70 [0.63, 0.77] | 58 (53/91) [48, 68] | 84 (105/125) [77, 90] |
|  | 90 keV | -0.183 | 0.7 [0.62, 0.77] | 50 (46/91) [40, 60] | 87 (109/125) [81, 93] |
|  | 100 keV | -0.181 | 0.69 [0.62, 0.77] | 50 (46/91) [40, 60] | 86 (108/125) [80, 92] |
|  | 110 keV | -0.177 | 0.69 [0.62, 0.76] | 49 (45/91) [39, 59] | 85 (107/125) [79, 91] |
|  | 120 keV | -0.142 | 0.69 [0.62, 0.76] | 46 (42/91) [35, 56] | 87 (109/125) [81, 92] |
|  | 130 keV | -0.102 | 0.69 [0.61, 0.76] | 44 (40/91) [33, 54] | 87 (110/125) [82, 93] |
|  | 140 keV | -0.098 | 0.68 [0.61, 0.76] | 44 (40/91) [33, 54] | 87 (110/125) [82, 93] |
|  | 150 keV | -0.107 | 0.68 [0.61, 0.76] | 44 (40/91) [33, 54] | 86 (108/125) [80, 92] |
|  | 160 keV | -0.122 | 0.68 [0.61, 0.75] | 46 (42/91) [35, 56] | 85 (107/125) [79, 91] |
|  | 170 keV | -0.176 | 0.68 [0.61, 0.75] | 51 (47/91) [41, 61] | 79 (100/125) [72, 86] |
|  | 180 keV | -0.177 | 0.68 [0.61, 0.75] | 51 (47/91) [41, 61] | 79 (100/125) [72, 86] |
|  | 190 keV | -0.125 | 0.68 [0.61, 0.75] | 46 (42/91) [35, 56] | 84 (106/125) [78, 90] |
|  | 200 keV | -0.176 | 0.68 [0.61, 0.75] | 51 (47/91) [41, 61] | 78 (99/125) [71, 86] |
| **Kurtosis** | |  |  |  |  |
|  | Conventional images |  |  |  |  |
|  | VMIs |  |  |  |  |
|  | 110 keV | 2.085 | 0.61 [0.54, 0.69] | 98 (90/91) [96, 10] | 30 (38/125) [22, 38] |
|  | 120 keV | 2.103 | 0.62 [0.55, 0.69] | 98 (90/91) [96, 10] | 30 (39/125) [22, 39] |
|  | 130 keV | 2.112 | 0.62 [0.55, 0.70] | 98 (90/91) [96, 10] | 33 (42/125) [25, 41] |
|  | 140 keV | 2.109 | 0.63 [0.56, 0.70] | 98 (90/91) [96, 10] | 33 (42/125) [25, 41] |
|  | 150 keV | 2.113 | 0.63 [0.56, 0.70] | 97 (89/91) [94, 10] | 33 (42/125) [25, 41] |
|  | 160 keV | 2.099 | 0.63 [0.56, 0.71] | 98 (90/91) [96, 10] | 32 (41/125) [24, 40] |
|  | 170 keV | 2.097 | 0.64 [0.56, 0.71] | 98 (90/91) [96, 10] | 32 (41/125) [24, 40] |
|  | 180 keV | 2.095 | 0.64 [0.57, 0.71] | 98 (90/91) [96, 10] | 32 (41/125) [24, 40] |
|  | 190 keV | 2.09 | 0.64 [0.57, 0.71] | 98 (90/91) [96, 10] | 32 (41/125) [24, 40] |
|  | 200 keV | 2.092 | 0.64 [0.57, 0.72] | 98 (90/91) [96, 10] | 31 (40/125) [23, 39] |
| **Variance** | |  |  |  |  |
|  | Conventional images | 14662.378 | 0.7 [0.63, 0.77] | 59 (54/91) [50, 67] | 78 (98/125) [69, 86] |
|  | VMIs |  |  |  |  |
|  | 40 keV | 69927.356 | 0.63 [0.56, 0.71] | 55 (50/91) [46, 63] | 74 (93/125) [65, 83] |
|  | 50 keV | 33790.222 | 0.69 [0.62, 0.76] | 64 (59/91) [56, 73] | 72 (91/125) [63, 81] |
|  | 60 keV | 27513.704 | 0.70 [0.63, 0.77] | 56 (51/91) [47, 64] | 81 (102/125) [73, 89] |
|  | 70 keV | 14634.876 | 0.70 [0.63, 0.77] | 64 (58/91) [55, 72] | 74 (93/125) [65, 83] |
|  | 80 keV | 12823.739 | 0.70 [0.63, 0.77] | 59 (54/91) [50, 67] | 79 (99/125) [70, 87] |
|  | 90 keV | 8064.392 | 0.71 [0.64, 0.77] | 68 (62/91) [59, 76] | 70 (88/125) [60, 79] |
|  | 100 keV | 6665.098 | 0.71 [0.64, 0.77] | 70 (64/91) [62, 78] | 68 (85/125) [58, 77] |
|  | 110 keV | 6449.416 | 0.70 [0.63, 0.77] | 68 (62/91) [60, 76] | 70 (88/125) [60, 79] |
|  | 120 keV | 5573.418 | 0.70 [0.63, 0.77] | 70 (64/91) [62, 78] | 68 (85/125) [58, 77] |
|  | 130 keV | 5268.949 | 0.70 [0.63, 0.77] | 70 (64/91) [62, 78] | 68 (85/125) [58, 77] |
|  | 140 keV | 5084.945 | 0.70 [0.63, 0.77] | 70 (64/91) [62, 78] | 68 (85/125) [58, 77] |
|  | 150 keV | 4425.313 | 0.70 [0.63, 0.77] | 74 (67/91) [66, 81] | 64 (81/125) [55, 74] |
|  | 160 keV | 4421.566 | 0.70 [0.63, 0.77] | 73 (67/91) [65, 81] | 65 (82/125) [56, 75] |
|  | 170 keV | 4306.755 | 0.70 [0.63, 0.77] | 74 (67/91) [66, 81] | 65 (82/125) [56, 75] |
|  | 180 keV | 4212.226 | 0.70 [0.63, 0.77] | 74 (67/91) [66, 81] | 65 (82/125) [56, 75] |
|  | 190 keV | 4128.979 | 0.70 [0.63, 0.77] | 74 (67/91) [66, 81] | 65 (82/125) [56, 75] |
|  | 200 keV | 4077.3 | 0.70 [0.63, 0.77] | 74 (67/91) [66, 81] | 65 (82/125) [56, 75] |
| **Uniformity** | |  |  |  |  |
|  | Conventional images | 0.042 | 0.89 [0.84, 0.93] | 84 (76/91) [77, 90] | 84 (106/125) [77, 92] |
|  | VMIs |  |  |  |  |
|  | 40 keV | 0.042 | 0.93 [0.89, 0.97] | 89 (82/91) [84, 95] | 90 (113/125) [84, 96] |
|  | 50 keV | 0.041 | 0.90 [0.86, 0.94] | 80 (73/91) [73, 87] | 86 (109/125) [79, 93] |
|  | 60 keV | 0.042 | 0.90 [0.86, 0.94] | 81 (74/91) [74, 88] | 86 (109/125) [79, 93] |
|  | 70 keV | 0.042 | 0.88 [0.84, 0.93] | 82 (75/91) [75, 89] | 81 (102/125) [73, 89] |
|  | 80 keV | 0.048 | 0.89 [0.85, 0.93] | 80 (73/91) [73, 87] | 86 (109/125) [79, 93] |
|  | 90 keV | 0.048 | 0.87 [0.82, 0.92] | 80 (74/91) [73, 87] | 83 (104/125) [75, 91] |
|  | 100 keV | 0.048 | 0.87 [0.82, 0.92] | 81 (74/91) [74, 88] | 83 (104/125) [75, 91] |
|  | 110 keV | 0.043 | 0.85 [0.79, 0.90] | 92 (84/91) [88, 97] | 67 (84/125) [57, 76] |
|  | 120 keV | 0.044 | 0.86 [0.81, 0.91] | 91 (84/91) [87, 96] | 70 (88/125) [60, 79] |
|  | 130 keV | 0.048 | 0.83 [0.77, 0.89] | 84 (77/91) [78, 90] | 72 (91/125) [63, 81] |
|  | 140 keV | 0.052 | 0.83 [0.77, 0.89] | 78 (71/91) [70, 85] | 80 (100/125) [72, 88] |
|  | 150 keV | 0.052 | 0.83 [0.77, 0.88] | 76 (70/91) [68, 83] | 80 (100/125) [72, 88] |
|  | 160 keV | 0.051 | 0.83 [0.78, 0.89] | 77 (70/91) [69, 84] | 79 (99/125) [70, 87] |
|  | 170 keV | 0.048 | 0.83 [0.78, 0.89] | 87 (79/91) [81, 92] | 71 (89/125) [62, 80] |
|  | 180 keV | 0.051 | 0.83 [0.78, 0.89] | 78 (72/91) [71, 86] | 75 (95/125) [67, 84] |
|  | 190 keV | 0.052 | 0.84 [0.79, 0.9] | 79 (73/91) [72, 86] | 78 (98/125) [69, 86] |
|  | 200 keV | 0.052 | 0.84 [0.79, 0.89] | 80 (73/91) [73, 87] | 78 (98/125) [69, 86] |
